# Linking Targeted GC–MS Disinfection Byproduct Analysis with Nontargeted LC–HRMS Characterization of Dissolved Organic Matter to Evaluate Drinking Water Treatment Performance

**DOI:** 10.1021/acsestwater.5c01470

**Published:** 2026-03-12

**Authors:** Francisco Zafra-Navarro, Saida Martí, Simeon Schum, Pere Emiliano, Mira Petrovič, Maria José Farré

**Affiliations:** † Catalan Institute for Water Research (ICRA), 17003 Girona, Spain; ‡ University of Girona (UdG), 17004 Girona, Spain; § 4423 New Mexico State University, Chemical Analysis and Instrumentation Laboratory (NMSU), Las Cruces, New Mexico 88003, United States; ∥ Ens d’Abastament d’Aigua Ter-Llobregat (ATL), 08970 Sant Joan Despí, Spain; ⊥ Catalan Institution for Research and Advanced Studies (ICREA), 08010 Barcelona, Spain

**Keywords:** adsorbable organic halides (AOX), chlorine disinfection, DBP precursors, formation potential test, gas chromatography−mass spectrometry (GC-MS), halogenated organic matter, molecular fingerprinting and water quality

## Abstract

Evaluating drinking water treatment (DWT) performance requires understanding how dissolved organic matter (DOM) is transformed during treatment and how these transformations drive the formation of disinfection byproducts (DBPs), which remain major concerns in drinking water safety. While current regulations target a limited number of DBP classes, including trihalomethanes (THMs) and haloacetic acids (HAAs), chlorination of DOM produces a much broader pool of largely unregulated DBPs with poorly understood toxicological relevance. In this study, a full-scale conventional drinking water treatment plant was evaluated using an integrated analytical framework combining molecular-level DOM fingerprinting by high-resolution mass spectrometry, targeted DBP quantification by gas chromatography–mass spectrometry, and adsorbable organic halide measurements. This integrated approach enables the assessment of DOM transformation and DBP formation under realistic treatment conditions. Although approximately 60% of dissolved organic carbon (DOC) was removed, molecular fingerprinting revealed a highly selective DOM removal pattern. Aromatic and condensed aromatic compounds were preferentially eliminated, whereas aliphatic and unsaturated fractions persisted and showed a positive statistical association with DBP formation. These results indicate that bulk DOC removal alone is insufficient to mitigate DBP formation and highlight the need for treatment strategies targeting specific reactive DOM fractions to enhance drinking water safety.

## Graphical abstract

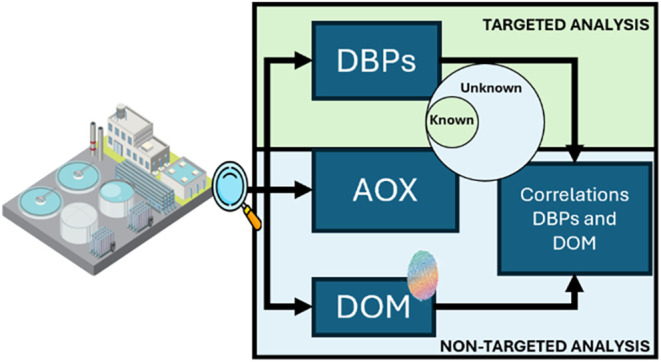

## 1 Introduction

Chlorination remains the most widely used disinfection method in drinking water treatment (DWT) due to its simplicity and cost-efficiency. However, reactions between chlorine-based disinfectants and dissolved organic matter (DOM) lead to the formation of disinfection byproducts (DBPs),^1,2^ which pose significant health risks due to their potential genotoxicity and carcinogenicity.^1,3^ In spite of more than 1000 DBPs having been identified to date,^4^ current regulatory frameworks, including the European Drinking Water Directive (2020/2184)^5^ and the U.S. National Primary Drinking Water Regulations (NPDWR),^6^ address only a limited number of halogenated DBPs, primarily trihalomethanes (THMs) and haloacetic acids (HAAs). This regulatory gap overlooks numerous unregulated DBP classes, which may collectively pose greater health risks than the regulated compounds alone.^7^

The structural diversity and wide physicochemical range of DBPs pose major analytical challenges. Targeted gas chromatography–mass spectrometry (GC-MS)^8^ and liquid chromatography–mass spectrometry (LC–MS)^9^ methods enable the quantification of selected DBPs but are inherently limited to predefined analytes. Adsorbable organic halogen (AOX) provides a bulk estimate of the total halogenated organic matter but lacks molecular-level information. In this context, non-targeted high-resolution mass spectrometry (HRMS) enables the detection and characterization of previously unidentified DBPs,^7^ offering a complementary approach for comprehensive DBP assessment.

On the other hand, DOM is the main precursor for DBP formation, yet its composition is highly variable, influenced by source water characteristics, seasonal dynamics, and catchment conditions.^10^ This variability affects DOM reactivity with disinfectants and complicates the prediction of DBP formation.^11^

Consequently, drinking water treatment technologies range from conventional processes such as coagulation and sand filtration^12^ to advanced methods including ultrafiltration or reverse osmosis,^13^ as well as widely used methods like activated carbon filtration.^14^ However, treatment performance is typically evaluated based on bulk parameters such as total organic carbon (TOC) removal, which do not necessarily reflect the elimination of the most reactive DBP precursors.^12^

DOM characterization employs a range of techniques with varying levels of molecular resolution. Bulk measurements such as TOC and dissolved organic carbon (DOC)^15^ quantify total organic content, while spectrometric methods provide information on DOM aromaticity,^16^ protein-like content,^17^ and aromatic moieties.^18^ However, these approaches offer limited molecular-level detail, a gap addressed by HRMS, which provides detailed insights into DOM composition, structural diversity, and reactivity.^7,19–22^

In particular, HRMS has been applied to investigate DOM transformations in DWTPs, focusing on either DBP formation or DOM removal.^23^ Most studies compare influent and effluent samples,^19^ while others examine the entire treatment train at full-scale DWTPs^24^ or explore DBP formation mechanisms through controlled laboratory experiments.^25^ However, few studies integrate multiple analytical approaches—including targeted DBP quantification, AOX measurement, and HRMS-based DOM profiling, within a unified framework to comprehensively link DOM dynamics and DBP formation across the complete treatment process. Even when integrated approaches are used, they often rely on influent/effluent comparisons,^22^ potentially overlooking DOM transformations at intermediate treatment stages that influence final DBP formation.

The present study evaluates a conventional DWTP using an integrated analytical framework linking DOM transformations with DBP formation under full-scale conditions. We combine (i) non-targeted HRMS for molecular fingerprinting (Figure 1); (ii) targeted quantification of 17 regulated and emerging DBPs by GC-MS (Figure 3); and (iii) AOX determination, including speciation into AOCl, AOBr, and AOI. This multitechnique approach enables a comprehensive investigation of how DOM removal efficiency and compositional shifts along the treatment train relate to DBP formation.

Importantly, it highlights which DOM fractions are most reactive to chlorination and which persist through treatment, clarifying whether reactive and poorly removed fractions overlap or represent distinct molecular pools. Such insights are essential for developing targeted treatment strategies that specifically address DBP precursor removal and enhance drinking water safety beyond conventional bulk parameter optimization.

## 2 Materials and Methods

### 2.1 Chemicals and Reagents

For the preparation of formation potential (FP) experiments, free and total chlorine concentrations were measured using commercial *N*,*N*-diethyl-*o*-phenylenediamine (DPD) test kits (LCK310, Hach Lange) and a Hach DR3900 spectrophotometer (Hach, USA). 5 M phosphate buffer solutions were prepared with potassium dihydrogen phosphate (KH_2_PO_4_, ≥99%, Sigma-Aldrich) and sodium hydroxide (NaOH, ≥99%, Sigma-Aldrich). Residual chlorine was quenched at the end of the FP tests using ascorbic acid (C_6_H_8_O_6_ ≥ 98%, Sigma-Aldrich).

For DBP analysis, haloacetonitrile (HAN) standards were obtained as a 5.0 mg/mL mixture in acetone (>95% purity, Cluzeau, Sainte-Foy-la-Grande, France); haloacetic acid (HAA) standards were provided as a 1.0 mg/mL mixture in methanol from Merck (Merck Life Science, Darmstadt, Germany); and trihalomethane (THM) standards were purchased as a 1.0 mg/mL mixture in methanol (TraceCERT grade, Sigma-Aldrich, USA). Deuterated 1,2-dibromopropane-*d*_6_ (99.6 atom % D, CDN Isotopes, Quebec, Canada) and d-chloroform (99.8 atom % D, Merck KGaA, Darmstadt, Germany) were used as the internal standards for DBP quantification. Additional reagents including methyl *tert*-butyl ether (MTBE, Chromasolv Plus, Sigma-Aldrich, USA), sodium hypochlorite solution (NaClO, 6–14% active chlorine, Emplura grade, Sigma-Aldrich, USA), and sulfuric acid (95–97%, Reag. Ph Eur grade, Scharlau, Spain) were used.

For AOX determination, nitric acid (HNO_3_, ≥70%), sodium nitrate (NaNO_3_, ≥99%, Sigma-Aldrich), and potassium hydroxide (KOH, ≥99%, Sigma-Aldrich) were obtained from Sigma-Aldrich (Missouri, USA). Ultrapure water (Optima LC/MS grade) was supplied by Fisher Chemical (Geel, Belgium).

For the analysis of DOM, ultrapure water and methanol (Optima LC/MS grade) were supplied by Fisher Chemical (Geel, Belgium, and Loughborough, U.K., respectively). Formic acid (98–100%, ACS grade) was obtained from Merck (Darmstadt, Germany). Nitrogen gas (99.995% purity) for extract drying was supplied by Abello Linde (Barcelona, Spain). Water samples were filtered using glass fiber filters (GF/F, 0.7 μm, 47 mm diameter, Whatman, U.K.). Suwannee River natural organic matter (SRNOM, 2R101N) reference material was acquired from the International Humic Substances Society (IHSS, Minnesota, USA). Solid-phase extraction (SPE) was conducted using Bond Elut PPL cartridges (styrene–divinylbenzene sorbent, Agilent Technologies, USA).

### 2.2 DWTP and Sampling

The drinking water treatment plant (DWTP) evaluated in this study is located in Barcelona, Spain, and is operated by Ens d’Abastament d’Aigua Ter-Llobregat (ATL). It supplies drinking water to the metropolitan area of Barcelona, serving a population of over 4.5 million inhabitants. During the monitoring period, the DWTP treated an average flow rate of approximately 2.85 m^3^/s. The influent water originated from a system of interconnected reservoirs (Sau–Susqueda–Pasteral) and was conveyed to the facility through a 56 km pipeline.^26^ Operational parameters recorded during each sampling campaign are listed in Table S1.

The treatment process follows a conventional multi-barrier design. It begins with primary disinfection, involving the sequential addition of chlorine dioxide and sodium hypochlorite. This is followed by coagulation and flocculation using polyaluminum chloride and modified starch, and subsequent sedimentation. The clarified water is then filtered by gravity through granular activated carbon (GAC). After filtration, the treated river water is blended with bromide-rich desalinated water and subjected to secondary disinfection using sodium hypochlorite. The final treated water is distributed through the municipal supply network to a system of service tanks located throughout the metropolitan area.

To assess the performance of the treatment system and the evolution of water quality throughout the distribution network, seven sampling points were selected: (1) Raw water: DWTP influent; (2) Clarified: after initial pre-oxidation with chlorine dioxide; (3) Post AC: after GAC filtration; (4) Produced water: treated river water blended with desalinated water and post-disinfection; (5) Distribution 24 h: stored in tanks with a hydraulic retention time (HRT) of 24 h; (6) Distribution 48 h: stored in tanks with an HRT of 48 h; and (7) Distribution 72 h: stored in tanks with an HRT of 72 h.

Samples were collected across different seasons to cover a range of hydrological conditions and reduce sampling bias; six sampling campaigns were performed during 2023 and 2024 in three seasons, spring (April 2023), autumn (November 2023), and winter (January 2024) with two sampling events per season. For each sampling point during all sampling events, routine physicochemical parameters were measured, including DOC, conductivity, pH, nitrite, nitrate, phosphate, chlorine, sulfate, bromide, fluoride, chlorite, chlorate, iodine, sodium, ammonia, total hardness, and total nitrogen (TN). The measured values and the quantification limits are provided in Tables S2 and S3.

Formation potential (FP) test was performed on the raw water sample to establish the potential formation of DBPs in the influent of the DWTP and to identify links between DOM fingerprinting and DBP formation. Details on how the FP tests were conducted are provided in the Supporting Information.

### 2.3 Target Analysis of DBPs Using GC-MS

Seventeen target DBPs, grouped into three families, THMs, HAAs, and HANs, were analyzed using a liquid–liquid salted microextraction followed by gas chromatography coupled to tandem mass spectrometry (GC-MS/MS). Specific details of the DBP extraction are provided in the Supporting Information.

### 2.4 AOX Analysis

AOX determinations were differentiated into chlorinated (AOCl), brominated (AOBr), and iodinated (AOI) halide ions. Their limits of detection were 1.85 μg/L for chloride (Cl^–^), 0.25 μg/L for bromide (Br^–^), and 0.71 μg/L for iodide (I^–^). More complete information is provided in the Supporting Information.

### 2.5 DOM Characterization Using LC-HRMS

A volume of 2 L of water was filtered through glass fiber filters (0.7 μm mesh size, Whatman). Samples were acidified to pH ≈2 using formic acid; pH was verified prior to SPE extraction, and samples were subsequently extracted using solid-phase extraction (SPE) with Bond Elut PPL cartridges (500 mg, 3 mL, Agilent Technologies). To determine extraction recovery for each sample, DOC was measured before and after SPE. Recovery values are summarized in Table S4. Finally, based on the DOC measurements, all extracts were subsequently diluted with methanol to achieve a standardized total carbon concentration of 1000 mg/L in each LC vial. This DOC normalization ensured that observed differences in the HRMS signal intensities reflected compositional changes rather than concentration effects.

The extracts were injected into a Vanquish UHPLC system coupled to an Orbitrap Exploris 120, equipped with a Hypersil GOLD C18 column and an electrospray ionization (ESI) source operating in negative-ion mode.

The HRMS spectra of DOM were processed using FreeStyle 1.5 software (Thermo Scientific). Mass lists, generated by averaging spectra over the retention time window of DOM (4–9 min),^21^ as shown in Figure S1, were employed for formula assignment.

Molecular formula assignment was performed in RStudio (v4.4.0) using a custom script based on the MFAssignR package,^27^ along with other relevant R packages (R Core Team, 2024). Elemental and structural limits were applied according to Hawkes et al.,^28^ including a maximum mass error of 1 ppm, O/C ratios from 0 to 1, H/C ratios from 0.3 to 2.5, DBE–O from −10 to 10, and molecular formula ranges of C_4_–_50_H_4_–_100_O_2_–_40_N_0_–_2_S_0_–_1_Cl_0_–_3_Br_0_–_3_.^28^

Only molecular formulas detected in at least 4 out of the 6 samples per DWTP stage were retained for van Krevelen representation and subsequent analysis (Table S5).^29^ This detection frequency filter was applied to balance sensitivity and robustness, prioritizing reproducible DOM features representative of treatment processes and suitable for correlation-based precursor analysis.^29^

Weighted averages of DBEw, O/Cw, H/Cw, and DBE-Ow were calculated for each DWTP stage following the approach of Maizel et al.,^30^ and the results are presented in Table S6.

For halogenated compounds containing ^35^Cl and ^79^Br, a secondary validation step was included. A custom R function cross-checked the isotopic patterns of assigned formulas by verifying the presence and relative abundance of the corresponding isotopic peaks (^37^Cl and ^81^Br), ensuring consistency with natural abundance ratios (tolerances: ±3 mDa for *m*/*z* and ±30% for isotopic ratios; Figure S2 and S3). This resulted in molecular formula assignments consistent with “molecular formula only” reporting (often aligned with Schymanski’s level 4 ^31^ in non-target workflows), without implying structural identification. The identified features were visualized using van Krevelen diagrams based on O/C and H/C ratios, following established van Krevelen region classifications commonly used for DOM interpretation (e.g., Kellerman et al.;^32^ Hawkes et al.^28^) Features were classified as aliphatic, aromatic, condensed aromatic, high-oxygen unsaturated, and low-oxygen unsaturated-like compounds according to the regions they occupy in the van Krevelen diagram. An example of this classification using the SRNOM standard is presented in Figure S4. The definitions of the categories are summarized in Table S7.

Furthermore, information about SPE extraction, LC injection, and molecular formula assignment is detailed in Supporting Information.

## 3 Results and Discussion

To comprehensively characterize the formation and behavior of halogenated organic matter throughout the drinking water treatment process, three complementary analytical approaches were applied to samples collected across the DWTP: (i) non-targeted HRMS for molecular fingerprinting (Figure 1); (ii) targeted quantification of 17 regulated and emerging DBPs by GC–MS (Figure 3); and (iii) determination of AOX, including the speciation of chlorinated (AOCl), brominated (AOBr), and iodinated (AOI) fractions (Figure 3).

This integrated framework provides a detailed molecular-level perspective on halogenated dissolved organic matter (X-DOM) and DBP formation, enabling simultaneous assessment of precursor reactivity, the generation of new halogenated species, and the selective removal of DOM fractions along the treatment train. Importantly, combining non-targeted and targeted analyses allows the evaluation of whether the DOM fractions that persist through treatment are those most prone to generate regulated DBPs—a critical aspect in optimizing treatment strategies aimed at minimizing harmful byproducts in drinking water.

### 3.1 Changes in the Fingerprint of DOM among the Treatment

Van Krevelen diagrams (Figure 1) were used to visualize compositional shifts in DOM along the DWTP, complemented by relative frequency and cumulative frequency distributions of intensity changes (Figure 2). Together, these tools enable a deeper interpretation of DOM reactivity and removal beyond bulk DOC measurements.

Figure 1A shows a clear overall depletion of DOM features, with the most pronounced reduction occurring in aromatic and condensed aromatic-like compounds. This pattern indicates that these fractions are efficiently removed by the treatment train. In contrast, Figure 1B reveals a marked increase in halogenated molecular features within the low-oxygen region, suggesting the in situ formation of new halogenated organic matter during treatment—consistent with previous observations.^20,33–34^

An enrichment in both non-halogenated and halogenated features, in the unsaturated region, is also observed after the activated carbon filtration step. This trend may reflect the preferential removal of high-molecular-weight DOM, resulting in an apparent enrichment of smaller, potentially more reactive species.^35^ The co-enrichment of halogenated features in the same region (Figure 1B) across all stages aligns with earlier studies reporting that DBP-correlated molecular features—including those associated with THMs, HAAs, and HANs—cluster predominantly within this van Krevelen region,^20,33,34^ suggesting that this halogenated matter could act as a reactive intermediate after chlorination and before the formation of regulated DBPs.^19,20,23,33,34,36^

**Figure 1:**
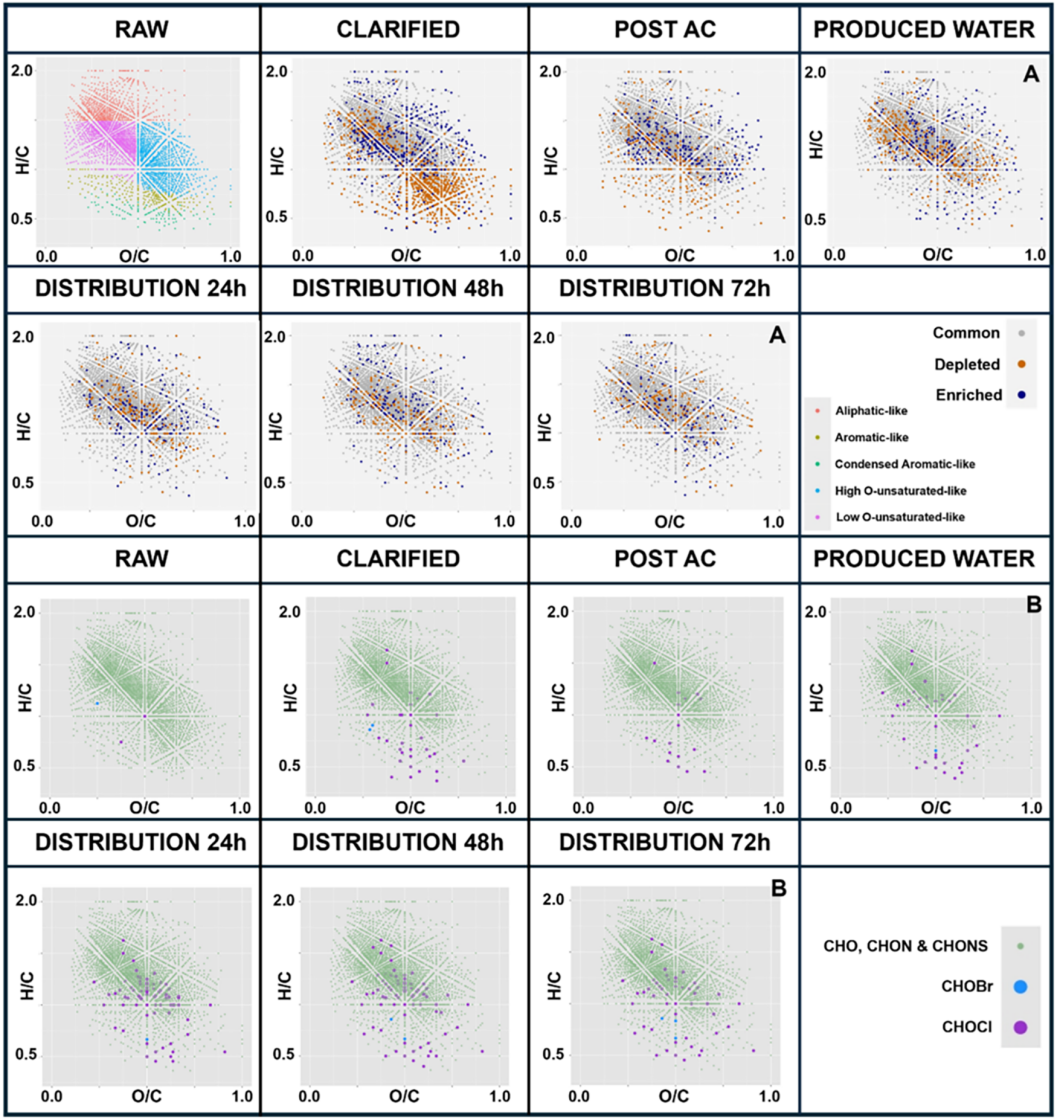
Mean relative changes in the molecular features detected across all samplings along the DWTP, highlighting depletion and enrichment trends based on features showing at least a 10-fold change in abundance compared to the previous treatment stage, where raw is represented in a Van Krevlen representation,^28,32^ with aliphatics, aromatics, condensed aromatics, high-oxygen unsaturated, and low-oxygen unsaturated compounds (A). Van Krevelen diagrams showing the distribution of molecular features at each DWTP sampling point, distinguishing halogenated from nonhalogenated compounds (B).

Figures 1 and 2 provide additional evidence that DOM removal is not uniform across molecular classes. While DOC removal between raw and treated water ranged from 41.6 to 75.2%, the relative intensity changes reveal that condensed aromatic-like compounds exhibit the greatest depletion, whereas high- and low-oxygen unsaturated fractions—typically linked to DBP formation—show comparatively little removal.^20,33,34^

A detailed quantification of the number of features across van Krevelen regions (Table S8) indicated removal ranges of 0–0.8%, 51–59%, 32–58%, 13–17%, and 5–11% for aliphatic, aromatic, condensed aromatic, high-oxygen unsaturated, and low-oxygen unsaturated features, respectively. Aromatic species appear to be removed primarily during pre-oxidation and coagulation–flocculation, consistent with their known high reactivity toward oxidants used in early DWTP stages.^35^ These steps thus contribute disproportionately to the overall DOM reduction.

In contrast, the aliphatic and unsaturated SPE-DOM fractions exhibited limited reductions across treatment steps, suggesting a higher persistence relative to aromatic fractions based on relative HRMS signal patterns under the applied analytical conditions. This selective persistence reinforces concerns that conventional treatment processes insufficiently target molecular fractions linked with DBP formation.^20,33,34^ This finding underscores the need for improved treatment strategies aimed at attenuating disinfectant reactivity and mitigating the formation of DBP.

### 3.2 Halogenated Organic Matter and Disinfection Byproducts

Figure 3 integrates the results of X-DOM, showing (A) DBP concentrations, (B) AOX profiles, and (C) the number of halogenated molecular features detected across the van Krevelen regions.

Of the 17 DBPs analyzed, 12 were detected: four THMs, three HANs, and five HAAs. In produced water, concentrations ranged from 13–42 μg/L for THMs, 1–4 μg/L for HANs, and 6–13 μg/L for HAAs. As expected, FP tests yielded considerably higher concentrations: 51–64 μg/L for THMs, 7–9 μg/L for HANs, and 56–63 μg/L for HAAs.

Comparing potential DBP formation (from FP samples) with actual concentrations (in produced water samples) reveals that the treatment process mitigates 34–75% of THM formation, 69–83% of HANs formation, and 79–89% of HAAs formation. These findings indicate that THM precursors remain the most challenging to remove, consistent with previous studies reporting THM precursor fractions in conventional treatment systems.^20,33,34^ Detailed concentration profiles for individual DBPs are shown in Figure S5 and Table S9.

**Figure 2:**
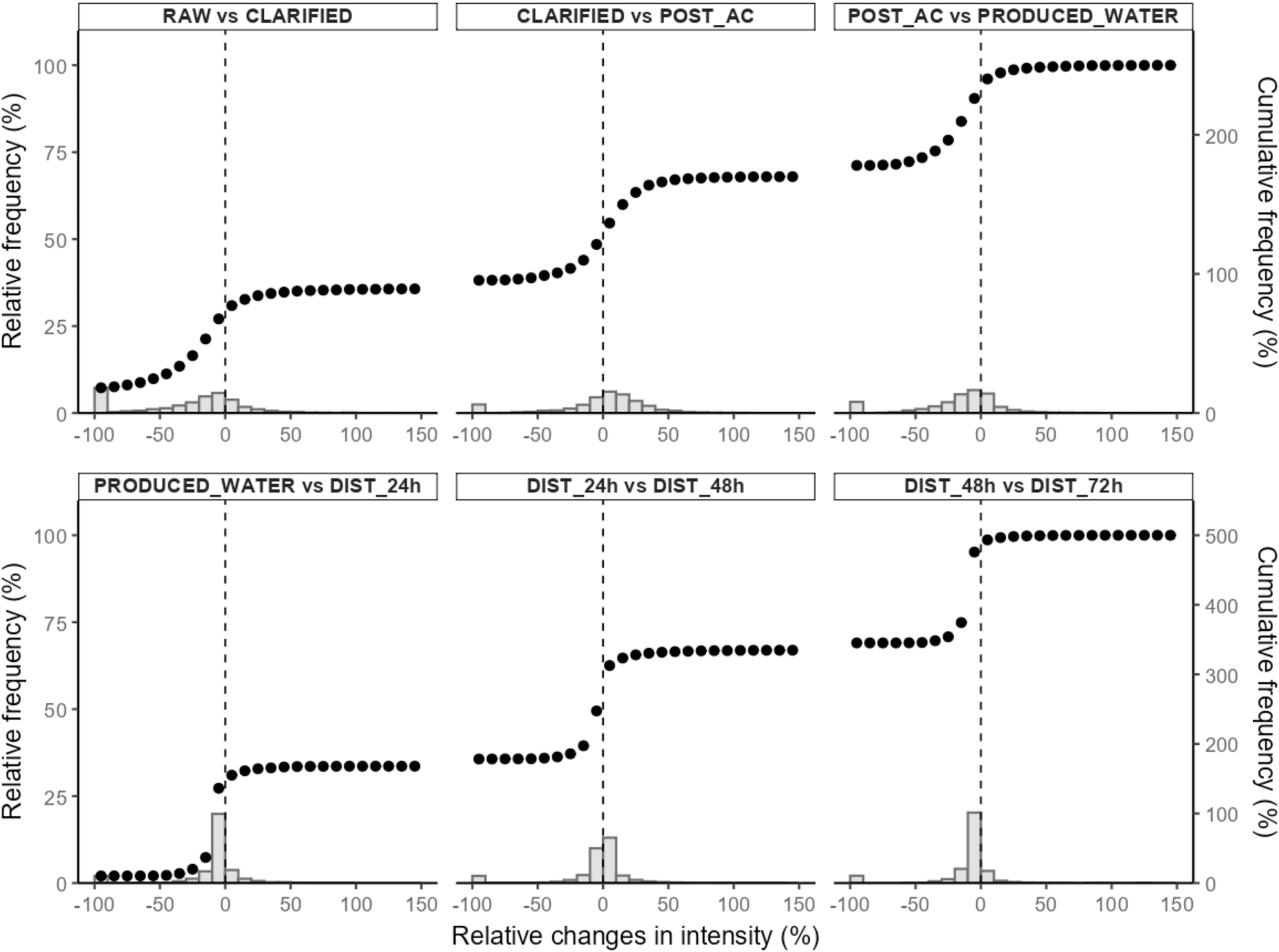
Relative frequency and relative cumulative frequency distributions of intensity changes between two sites (i.e., (Intensity site_n_ – Intensity site_n–1_)/Intensity site_n–1_) for the DWTP sampling points.

AOX analyses were performed only on disinfected samples (PW and D1–D3). AOCl and AOBr appeared to increase during distribution (Figure 3B); however, these changes were not statistically significant (*p* = 0.077 and 0.478, respectively). This indicates that the overall concentration of halogenated organic matter does not change substantially along the distribution system. Even so, a reduction in the number of halogenated features was observed in the HRMS data, while DBP concentrations continued to increase. This pattern reveals the potential role of halogenated organic matter as a reactive intermediate in the formation of smaller DBPs, such as THMs.

Comparing targeted DBPs with AOX, quantified DBPs accounted for less than 31% of total AOX in DWTP samples (Figure S6). These values align with previous studies reporting that less than 50% of AOX is typically explained by known DBPs in chlorinated water.^36^ This confirms that a substantial fraction of halogenated organic matter remains uncharacterized and highlights the need to integrate non-target approaches, such as HRMS, to accurately assess DBP-related risks.

HRMS fingerprinting (Figure 3C) revealed a biphasic pattern in the halogenated molecular features. A first increase occurred after pre-oxidation, particularly in aromatic-like regions, followed by partial removal during activated carbon filtration. Removal, however, was incomplete; after final chlorination, a second—and more pronounced—formation pulse was observed. With the exception of aliphatic-like compounds, most regions showed declining halogenated feature counts after disinfection, while DBP concentrations—particularly THMs—continued to increase during distribution (Figure 3A). This indicates that X-DOM persistence detected by HRMS is associated with ongoing DBP formation, especially under prolonged disinfectant contact.

#### 3.2.1 Chlorinated Dissolved Organic Matter

A total of up to 115 Cl-DOM features were detected (maximum at distribution 24h water samples), representing less than 3.1% of all DOM features. Despite this small fraction, Cl-DOM is of concern due to its established toxicity.^2^ An example HRMS spectrum illustrating the characteristic chlorine isotopic pattern of a representative Cl-DOM feature is shown in Figure S7. Cl-DOM first appeared after preoxidation (clarified samples) and increased further after main chlorination. Its distribution spans all van Krevelen regions, with the highest abundance in the condensed aromatic and high-oxygen unsaturated regions, and the lowest in aliphatic and aromatic-like regions.

Importantly, most Cl-DOM was located in unsaturated regions, especially the high-oxygen unsaturated space—a fraction poorly removed by the DWTP (less than 17%). Aromatic Cl-DOM was formed primarily during pre-oxidation rather than during final chlorination, likely due to the faster reaction kinetics of aromatic DOM with chlorine. Early formation can be advantageous, as activated carbon removes aromatic halogenated compounds more efficiently.^37^ However, Figure 3C shows that this removal is incomplete.

**Figure 3:**
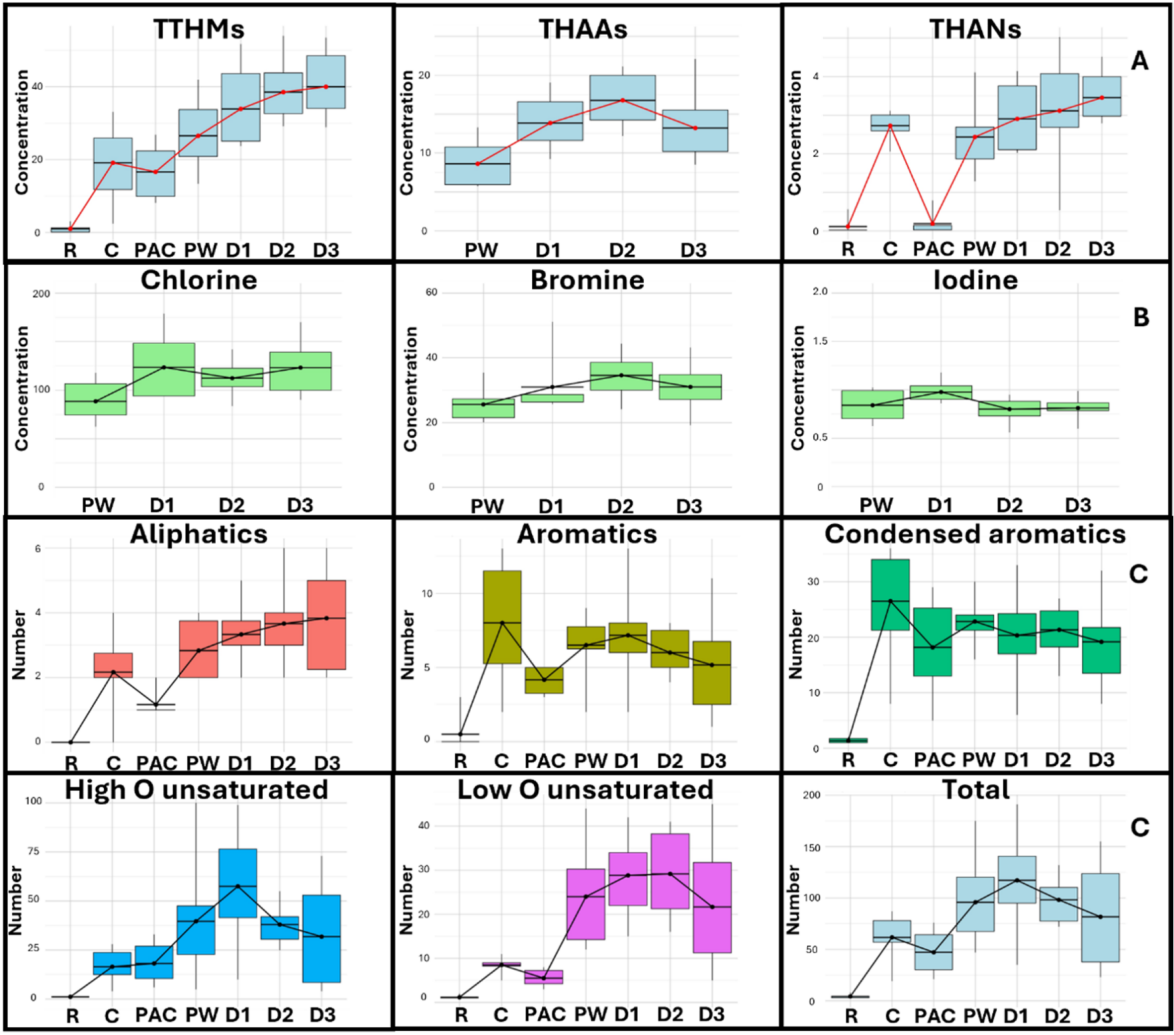
Concentration profiles of halogenated species using multiple analytical approaches: (A) target DBP analysis in actual samples (μg/L); (B) AOCl, AOBr and AOI in actual samples (mg/L); (C) number of halogenated molecular features detected by non-target HRMS and classified across van Krevelen regions. Feature counts represent aggregate halogenation patterns and do not imply the persistence of individual molecular identities across treatment stages. Changes in feature numbers should therefore be interpreted together with compositional and intensity-based analyses (Figures 1 and 2). DWTP points sampled and presented in this figure are coded as follows: R = Raw, C = Clarified, PAC = Post-activated carbon filtration, PW = Produced water, D1 = Distribution system after 24 h, D2 = Distribution system after 48 h, and D3 = Distribution system after 72 h.

Brominated DOM (Br-DOM) features were detected only sporadically and at very low frequency in this system, preventing a robust compositional or statistical assessment for this family of compounds. This limited occurrence is consistent with the moderate blending ratio of desalinated water (between 10 and 15%, Table S1) and the strong dominance of chloride over bromide during chlorination, which favors the formation of chlorinated over brominated DOM at the molecular fingerprint level. Consequently, Br-DOM was not discussed further to avoid overinterpretation. Although brominated DBPs were detected by targeted GC–MS/MS, nontarget LC–HRMS primarily captures dominant DOM compositional patterns and is not optimized for trace-level halogen speciation, explaining the apparent discrepancy between targeted and non-target results. Future studies with higher bromide levels or targeted experimental designs will be required to assess the impact of desalinated water blending on Br-DBP speciation.

### 3.3 DBPs Precursor Dynamics

The conventional DWTP evaluated in this study removes up to 75.2% of the DOC under optimal conditions. However, the molecular-level DOM fingerprint reveals that this reduction is largely associated with aromatic and condensed aromatic regions, where removal reaches up to 59%. In contrast, minimal removal was observed for aliphatic and unsaturated-like compounds. This incomplete elimination of specific DOM fractions appears to contribute to halogenated DBP formation, particularly THMs, which have previously been linked to these persistent DOM features.^20,33,34^

To investigate the relationship between DOM composition and DBP formation—and to determine whether the DOM fractions remaining after treatment contribute to DBP formation—Spearman rank correlation coefficients (ρ) were calculated between HRMS-derived molecular features of raw water samples and FP DBP concentrations. These HRMS-derived features are interpreted as DOM molecular fingerprints rather than structurally identified individual compounds and therefore represent reactive DOM components or isomeric ensembles. Only features detected in at least four of the six samples were included. These features were correlated with DBP concentrations measured under FP conditions, and those showing positive correlations with high ρ values (ρ > 0.8) were retained and are shown in Figure 4.

**Figure 4:**
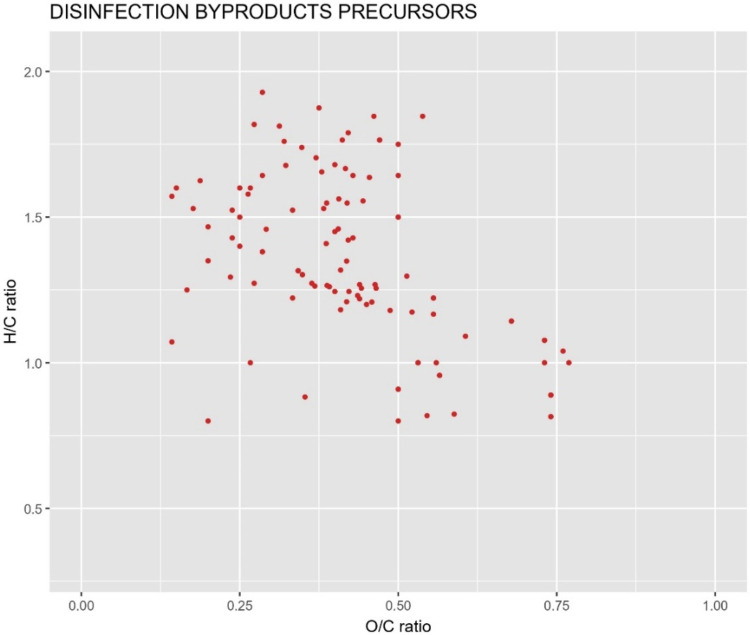
HRMS-derived DOM features in raw water showing high positive correlations (ρ > 0.8) with DBP formation potential.

A total of 97 features in raw water were associated with DBP formation (ρ > 0.8). These associations reflect statistical links between DOM molecular features and DBP formation potential rather than direct identification of individual precursor compounds. Their distribution across van Krevelen regions was: 38 aliphatic, 3 aromatic, 18 high-O unsaturated, and 38 low-O unsaturated-like compounds. This distribution suggests that aliphatic and unsaturated fractions may be more strongly associated with DBP formation than aromatic fractions in this system.

To evaluate the removal efficiency of these potential precursors, their presence across all treatment steps was assessed using the same detection frequency criterion (≥4 of 6 samples). As summarized in Table S10, 61 of the 97 tentative precursors were detectable after activated carbon filtration, the final step before disinfection. Thus, 37% of these indicative DBP precursor features were no longer detected after treatment, indicating complete removal due to the treatment.

Importantly, the remaining precursors were not necessarily persistent at constant levels. As shown in Figure S8, many exhibited partial attenuation or intensity changes across treatment steps, resulting in incomplete removal or transformation rather than full persistence. The majority of the precursors detected prior to disinfection were aliphatic-like (57%) and low-O unsaturated-like (31%), consistent with the trends described in Section 3.1, where these regions also showed the lowest overall removal efficiencies.

To gain a deeper understanding of the behavior of these potential precursors, the relative intensity changes of each precursor throughout the treatment are illustrated in Figure S8. Three distinct behavioral patterns were identified:

(i) Removal through treatment (31 precursors). These features showed progressive decreases in abundance, particularly those classified in the low-O unsaturated region (22 features). Their attenuation is likely driven by pre-oxidation and coagulation processes.
(ii) Enrichment after disinfection (48 precursors). For nearly half of the precursors, higher intensities were observed after disinfection. These were predominantly aliphatic (22 features) and unsaturated (24 features). Their apparent formation may result from the oxidative breakdown of larger DOM molecules into smaller species.
(iii) Formation after activated carbon filtration (15 precursors). A smaller subset showed increases after activated carbon filtration, including aliphatic (7) and high-oxygen unsaturated (6) features. This enrichment is consistent with observations in Section 3.1, suggesting that activated carbon preferentially removes high-molecular-weight DOM, leading to an apparent shift toward lower-molecular-weight species, which may be more reactive.

These results highlight the importance of more selective treatment strategies targeting the specific DOM fractions that act as DBP precursors. Most of the features showing positive correlations (ρ > 0.8) were classified as aliphatic and unsaturated-like compounds—groups that exhibited limited removal under the current treatment conditions. Although activated carbon is an effective step for aromatic and hydrophobic DOM, it is insufficient to eliminate the precursors that drive the formation of halogenated DBP in this system. Moreover, the formation of new reactive features after activated carbon underscores the need for a post-treatment step capable of removing low-molecular-weight and unsaturated DOM species that remain highly reactive toward disinfectants.

It is important to note that the correlation-based analysis presented here is exploratory and constrained to the SPE-extractable and ESI-negative-ionizable fractions of DOM accessed by the HRMS workflow. As a result, the associations identified reflect compositional domains within the measurable DOM pool rather than definitive molecular precursors. It should be noted that DOM is one of the most heterogeneous natural mixtures and that highly polar or otherwise non-extractable fractions may not be captured by a single analytical methodology such as SPE–ESI–HRMS.

### 3.4 Environmental Implications

In this study, a conventional DWTP with limited technological complexity was assessed using a multi-analytical approach, including the quantification of 17 target DBPs, the measurement of AOX, and DOM fingerprinting by HRMS.

The results show that while conventional treatment effectively reduces bulk DOC, DOM removal is highly selective. Aromatic and condensed aromatic-like fractions were efficiently eliminated, whereas aliphatic, low- and high-oxygen unsaturated regions persisted through the treatment train. These persistent fractions showed positive associations with the formation of halogenated organic features and regulated DBPs, particularly THMs. Spearman correlation analysis further indicated that regions with lower removal tended to correspond to compositional domains statistically linked to DBP formation.

These findings indicate that changes in the composition of the SPE-DOM fraction (captured by ESI(−)-HRMS) were more associated with DBP formation in this system. Even at relatively low DOC levels, unsaturated and aliphatic fractions appeared to play a significant role in the formation of DBP in this system. In systems with higher organic loads, the limited capacity of conventional processes to selectively remove these precursors poses a significant challenge for DBP control.

Importantly, the fractions that persist through treatment, primarily aliphatic and unsaturated regions, are the same fractions that are linked to DBP formation, highlighting an overlap between persistent DOM fractions and those statistically linked to DBP formation.

This study underscores the value of integrating multi-analytical approaches (GC–MS, AOX, HRMS) to comprehensively characterize X-DOM dynamics. The findings emphasize the need for selective or advanced treatment strategies capable of removing low-molecular-weight and unsaturated DOM, which is crucial for minimizing DBP formation while maintaining effective disinfection in drinking water systems.

## Supplementary Material



## Notes

Experimental details for disinfection byproduct formation potential tests; target disinfection byproduct analysis by GC–MS/MS, including limits of detection and quantification; adsorbable organic halogen (AOX) analysis; solid-phase extraction and LC–HRMS characterization of dissolved organic matter (DOM); DOM molecular formula assignment and data processing procedures; and tables and figures (Tables S1–S10 and Figures S1–S8) (PDF)
