# Supplementary material for: Linking Targeted GC–MS Disinfection Byproduct Analysis with Nontargeted LC–HRMS Characterization of Dissolved Organic Matter to Evaluate Drinking Water Treatment Performance
Source: ACS ES T Water. 2026 Mar 12;6(4):2458–67. doi: 10.1021/acsestwater.5c01470 (PMC13078287; doi:10.1021/acsestwater.5c01470)
Supplement: Supplementary file 1 [file ew5c01470_si_001.pdf]

Supplementary Information

"Linking Targeted GC–MS Disinfection Byproduct Analysis with Non-targeted LC–HRMS Characterization of Dissolved Organic Matter to Evaluate Drinking Water Treatment Performance."

Authors: Francisco Zafra-Navarro[1,2], Saida Martí[1], Simeon Schum[3], Pere Emiliano[4], Mira Petrovič[1,5] and Maria José Farré[1].

1Catalan Institute for Water Research (ICRA), 17003 Girona, Spain

2University of Girona (UdG), 17004 Girona, Spain.

3New Mexico State University; Chemical Analysis and Instrumentation Laboratory (NMSU), 88003 Las Cruces, United States.

4Ens d'Abastament d'Aigua Ter-Llobregat (ATL), 08970 Sant Joan Despí, Spain.

5Catalan Institution for Research and Advanced Studies (ICREA), 08010 Barcelona, Spain.

S1. Materials and methods.

S1.1 Formation Potentials.

The DBP formation potential (FP) was assessed following a modified version of the standard method (APHA, 2005) to obtain a residual free chlorine concentration between 1 and 3 mg/L after 72 hours of contact time, as described in previous studies[1–4]. A specific chlorine dose was added to a 2000 mL glass bottle according to the specific chlorine demand of each sample, ensuring no headspace. The bottle was then sealed and stored in an incubator at 25 °C in the dark for 72 hours.

After the 72-hour incubation period, the residual chlorine concentration was measured using a photometric cuvette test kit. After chlorine measurement, samples were quenched as suggested by Kristiana et al.[5] and as applied in previous studiefarr[3,6–16]. Quenching was performed with ascorbic acid solution (5 mg L^{-1} of ascorbic acid per mg L^{-1} of free chlorine) for DBP analysis (see Section S.2.2).

S1.2 Target analysis of DBPs using GC-MS.

DBP analysis was performed using liquid–liquid salted microextraction followed by gas chromatography (GC Trace 1300, Thermo Fisher Scientific) coupled to a triple quadrupole mass spectrometer (GC–MS/MS, Thermo Fisher Scientific). The analysis was conducted on a TSQ9000 triple quadrupole mass spectrometer (Thermo Fisher Scientific), equipped with a TriPlus™ autosampler.

The liquid–liquid salted microextraction procedure began with pH adjustment to 3.5 using 0.2 N sulfuric acid, followed by extraction with 3 mL of MBE containing 100 µg/L of d_6-1,2-dibromopropane and 150 µg/L of d-chloroform as internal standards. Subsequently, ~10 g of high-purity sodium sulphate was added, and the samples were vortexed for 1 minute before being left to settle for 5 minutes. Finally, ~1.5 mL of the MtBE extract was transferred into 2 mL vials for injection.

A 2 µL volume of extract was injected into GC-MS/MS in splitless mode (splitless time: 1 min) at 200 °C, onto a TG-1701MS column (30 m × 0.25 mm × 0.25 µm, Thermo Scientific). Chromatographic separation was performed using the following oven temperature program: 35 °C for 5 min, ramped to 100 °C at 10 °C/min, then further ramped to 200 °C at 20 °C/min and held for 1 min. Data acquisition and processing were performed using TraceFinder EFS 3.1 software. Retention times, SRM transitions, and GC–MS parameters used for DBP quantification have been described previously[3,17].

The DBPs analyzed were grouped into three families: THMs, HAAs, and HANs. A summary of the analyzed DBPs is provided in Table 1. The limits of detection (LOD) and quantification (LOQ) for each DBP are listed in Table 2.

Table 1.

List of disinfection byproducts analysed.

Family	Name	Abbreviation
Trihalomethanes	Chloroform	TCM
Trihalomethanes	Bromodichloromethane	BDCM
Trihalomethanes	Dibromochloromethane	DBCM
Trihalomethanes	Bromoform	TBM
Haloacetic acids	Bromoacetic acid	MBAA
Haloacetic acids	Chloroacetic acid	MCAA
Haloacetic acids	Dibromoacetic acid	DBAA
Haloacetic acids	Dichloroacetic acid	DCAA
Haloacetic acids	Trichloroacetic acid	TCAA
Haloacetic acids	Dibromochloroacetic acid	DBCAA
Haloacetic acids	Bromochloroacetic acid	BCAA
Haloacetic acids	Bromodichloroacetic acid	BDCAA
Haloacetic acids	Tribromoacetic acid	TBAA
Haloacetonitriles	Dichloroacetonitrile	DCAN
Haloacetonitriles	Trichloroacetonitrile	TCAN
Haloacetonitriles	Bromochloroacetonitrile	BCAN
Haloacetonitriles	Dibromoacetonitrile	DBAN

Table 2.

Limits of detection and quantification in µg/L of DBPs measured in the DWTP and FP samples.

Disinfection byproduct	LOD	LOQ
Chloroform (TCM)	0.15	0.49
Bromodichloromethane (BDCM)	0.03	0.11
Dibromochloromethane (DBCM)	0.01	0.05
Bromoform (TBM)	0.02	0.05
Bromoacetic acid (MBAA)	0.08	0.25
Chloroacetic acid (MCAA)	0.09	0.29
Dibromoacetic acid (DBAA)	0.02	0.07
Dichloroacetic acid (DCAA)	0.02	0.05
Trichloroacetic acid (TCAA)	0.01	0.03
Dibromochloroacetic acid (DBCAA)	0.05	0.18
Bromochloroacetic acid (BCAA)	0.03	0.10
Bromodichloroacetic acid (BDCAA)	0.01	0.04
Tribromoacetic acid (TBAA)	0.40	1.32
Dichloroacetonitrile (DCAN)	0.01	0.03
Trichloroacetonitrile (TCAN)	0.01	0.03
Bromochloroacetonitrile (BCAN)	0.01	0.03
Dibromoacetonitrile (DBAN)	0.03	0.10

S1.3 AOX analysis.

For AOX analysis, water samples were acidified to pH 2 using 0.2 M nitric acid/sodium nitrate solution (HNO_3/$NaNO_3$) before loading 100 mL of sample onto two consecutive activated carbon cartridges (40 mg activated carbon per glass column, 2 mm inner diameter; CPI International, Santa Rosa, California) using a Mitsubishi TXA-04 adsorption module (Tokyo, Japan).

Following enrichment, the cartridges were washed with 30 mL of 0.01 M nitrate solution (as $NaNO_3$) to remove inorganic halides. The activated carbon and adsorbed AOX were combusted in a Mitsubishi AQF-2100H automated furnace unit under an oxygen atmosphere at 1000 °C for 720 seconds. The pyrolysis gases generated, containing AOX in the form of acid halides (HX), were collected in a Mitsubishi GA-210 absorption unit containing 10 mL of absorption solution (ultrapure water).

To verify the complete removal of inorganic halides during the washing step, blank and spiked samples were analyzed following the same procedure. The absence of residual inorganic chloride in the AOX fraction was confirmed by ion chromatography analysis of the washing solution and blank cartridges.

Subsequently, 1000 µL of the absorption solution was injected into a Dionex Integrion HPIC (Thermo Fisher Scientific, Waltham, Massachusetts). Chromatographic separation was performed using a Dionex IonPac AS11-HC column (Thermo Fisher Scientific, Waltham, Massachusetts) with an IonPac AG11-HC guard column. The eluent was generated using a Dionex Eluent Generator Cartridge III with 30–75 mM KOH at a flow rate of 1 mL/min.

The limits of detection (LOD) for halide ions were 1.85 µg/L for chloride (Cl^-), 0.25 µg/L for bromide (Br^-), and 0.71 µg/L for iodide (I^-).

S2. DOM characterization using LC-HRMS.

S2.1 SPE extraction.

Water samples and procedural blanks for DOM characterization were filtered and extracted following established protocols described elsewhere[1,2,18]. A volume of 2 L of water was filtered through glass fibre filters (0.7 µm mesh-size, Whatman), acidified to pH 2.0 using approximately 5 mL of formic acid, and subsequently extracted using solid-phase extraction (SPE) with Bond Elut PPL cartridges (500 mg, 3 mL, Agilent Technologies).

Prior to extraction, the cartridges were activated by rinsing them three times with 1 mL of methanol and then soaking them in methanol for two hours. After activation, cartridges were conditioned with 3 mL of 0.1% (v/v) formic acid in ultrapure water to prepare them for sample loading. Acidified water samples (2 L, pH adjusted to 2.0 with formic acid) were then loaded onto the cartridges under vacuum at a flow rate of approximately 10 mL/min. Once the entire sample volume had passed through, cartridges were washed with 3 mL of 0.1% formic acid to remove loosely bound material. The drying process involved applying vacuum for 40 minutes, followed by an additional 15 minutes of drying under a stream of nitrogen to ensure complete removal of residual water. Finally, DOM was eluted with 2 mL of methanol, applying gentle pressure to the cartridge to maximize solvent recovery. Extracts were collected in pre-weighed LC vials for subsequent analysis.

To determine extraction recovery for each sample, DOC was measured before and after SPE. Prior to extraction, an aliquot of the sample was directly analyzed using a TOC-V CSH analyzer (Shimadzu). After SPE, a 300 µL aliquot of the extract was transferred to a clean test tube, and methanol was evaporated under a gentle nitrogen stream. The resulting dry residue was reconstituted in 10 mL of ultrapure water and analyzed. In addition, procedural blanks of the SPE extracts were prepared to ensure that no contamination occurred during sample reconstitution, as described by Li et al.[19]. Recovery values obtained from comparison of DOC measurements before and after SPE for each sample are summarized in Table S4. Based on the DOC measurements, all extracts were subsequently diluted with methanol to achieve a standardized total carbon concentration of 1000 mg/L in each LC vial, ensuring consistency for HRMS analysis.

S2.2 LC-HRMS instrumental analysis.

High-resolution mass spectrometric (HRMS) analyses were performed using an Orbitrap Exploris 120 (Thermo Scientific, Bremen, Germany) coupled to a Vanquish UHPLC system (Thermo Fisher Scientific), equipped with a Hypersil GOLD™ C18 column (50 mm × 2.1 mm, 1.9 µm, Thermo Scientific) and an electrospray ionization (ESI) source operating in negative-ion mode. SPE extracts were injected in methanol (10 µL). Although injection in 100% methanol can affect chromatographic retention, HRMS data were consistently evaluated within the DOM retention window (4–9 min; Figure S1) under the LC conditions reported in Tables 3 and 4.

Data acquisition was performed in full-scan mode over an m/z range of 200–1000, with a resolution of 120 000 FWHM (full width at half maximum). A total of 1000 scans were co-added for each mass spectrum. Methanol blanks were injected between samples to prevent carryover and cross-contamination, with one blank run between each sample. Fluoranthene (m/z 201.07825, $[C_{16}H_9]^-$) was systematically used as an internal calibrant by the Orbitrap Exploris 120 during HRMS analysis.

For data processing and molecular formula assignment, only mass signals detected between 4 and 9 minutes—corresponding to the retention time window of DOM in the chromatogram, as shown in Figure S1—were retained for analysis[9]. These masses were compiled into a dataset, and prior to formula assignment, the signals at m/z 369.08272 ($[C_{16}H_{17}O_{10}]^-$), 397.11402 ($[C_{18}H_{21}O_{10}]^-$), and 423.12967 ($[C_{20}H_{23}O_{10}]^-$) were monitored to ensure mass accuracy and instrument stability during injection[20,21].

Table 3.

LC separation conditions.

Time	Flow (mL/min)	%ACN+0.1%FA	Curve
0.0		Run	
3.0	0.3	1.0	5.0
12.0	0.3	99.0	5.0
14.0	0.3	99.0	5.0
15.0	0.3	1.0	5.0
17.0		Stop run	

Table 4.

HRMS conditions.

Ion Source Type	H-ESI
Spray Voltage	Static
Positive ion (V)	3500
Negative ion (V)	3500
Gas Mode	Static
Sheath Gas (Arb)	10
Aux Gas (Arb)	5
Sweep Gas (Arb)	0
Ion transfer tube temp (ºC)	325
Vaporizer temp (ºC)	350
APPI lamp	Not in use
FAIMS mode	Not installed

S2.3 Data processing: Formula assignment.

The HRMS spectra of DOM were processed using the software FreeStyle™ 1.5 (Thermo Scientific). Mass lists were generated by averaging spectra over the retention time window of DOM (4–9 min) and exported as Excel workbook files (.xlsx) for subsequent data analysis. This procedure yields a single entry per m/z value, thereby avoiding duplicate assignments arising from the chromatographic peak profile. Molecular formula assignment was performed in RStudio (v4.4.0) using a custom script based on the MFAssignR package[22], along with other relevant R packages (R Core Team, 2024).

To minimize noise, the KMDNoise function from MFAssignR was applied individually to each spectrum, leveraging Kendrick mass defect (KMD) plots to identify and estimate baseline signal intensity in regions where true ion signals were unlikely to occur. Peaks with an intensity greater than six times the estimated noise level were selected for molecular formula assignment using the MFAssign function.

Molecular formula assignment was constrained to include combinations of common elements found in natural organic matter (^{12}C, ^{1}H, ^{16}O) and selected heteroatoms (^{14}N, ^{32}S, ^{35}Cl, ^{79}Br). The assignment followed established chemical rules, such as the nitrogen rule, hydrogen deficiency (double bond equivalent, DBE), and DBE–O constraints.[10] Elemental and structural limits were applied according to Hawkes et al.,[23] including a maximum mass error of 1 ppm, O/C ratios from 0 to 1, H/C ratios from 0.3 to 2.5, DBE–O from –10 to 10, and molecular formula ranges of $C_{4-50}H_{4-100}O_{2-40}N_{0-2}S_{0-1}Cl_{0-3}Br_{0-3}$. Using the SRNOM reference standard as a quality control case, within the DOM retention

time window (4–9 min), a total of 6,771 mass features fulfilled the criteria for molecular formula assignment. Of these, 4,026 features (59.4%) received unambiguous molecular formula assignments, while no ambiguous assignments were retained. The mass error distribution was centered close to zero and remained within the applied ±1 ppm tolerance, confirming the typical mass accuracy achieved by the Orbitrap Exploris 120 (Figure S3).

The molecular formulae used in the van Krevelen representations were those detected in at least 4 out of the 6 samples per DWTP step and are summarized in Table S5. The weighted averages of DBE_w, O/C_w, H/C_w, and DBE–O_w were calculated for each DWTP stage as established by Maizel et al., [16] and the results are presented in Table S6.

For halogenated compounds containing ^{35}Cl and ^{79}Br, a secondary validation step was included. A custom R function cross-checked the isotopic patterns of assigned formulas by detecting ^{37}Cl and ^{81}Br isotopologues, ensuring consistency with natural abundance ratios (tolerances: ±3 mDa for m/z and ±30% for isotopic ratios). This resulted in a formula assignment with Level 4 confidence according to the Schymanski classification[23].

All samples were injected in duplicate. Only molecular formulas that were assigned in both replicates were retained to increase confidence in the assignments and to eliminate potential false positives. In addition, molecular formulas detected in the SPE procedural blanks were subtracted from those of the samples.

The identified features were visualized using van Krevelen diagrams based on O/C and H/C ratios, following established van Krevelen region classifications commonly used for DOM interpretation (e.g., Kellerman et al.[24]; Hawkes et al.[25]). Features were classified as aliphatic, aromatic, condensed aromatic, high-oxygen unsaturated, and low-oxygen unsaturated-like compounds according to the regions they occupy in the van Krevelen diagram. An example of this classification using SRNOM standard is presented in Figure S4. The definitions of the categories are summarized in Table S7.

To evaluate the performance of the formula-assignment pipeline, a quality control analysis was conducted using the SRNOM reference standard. The assigned formulas were compared against those available in publicly accessible datasets included in the InterLabStudy package (version 0.0.1.5)[26]. This dataset compiles SRNOM molecular formulas obtained from various HRMS analyses worldwide, encompassing both Orbitrap and FT-ICR-MS instruments. On average, 85% of the formulas assigned from our Orbitrap Exploris 120 data matched those in the InterLabStudy dataset, demonstrating the reliability and accuracy of our processing workflow. To further assess the non-matching fraction (15%), we compared matched and non-matched formulas in terms of chemical space (van Krevelen distribution), molecular mass, and signal intensity (Figure 1). Non-matching formulas largely overlapped with matched features in van Krevelen space and showed no clear compositional bias, and were predominantly associated with lower signal intensities, suggesting that differences are mainly driven by detection sensitivity rather than systematic instrument bias.

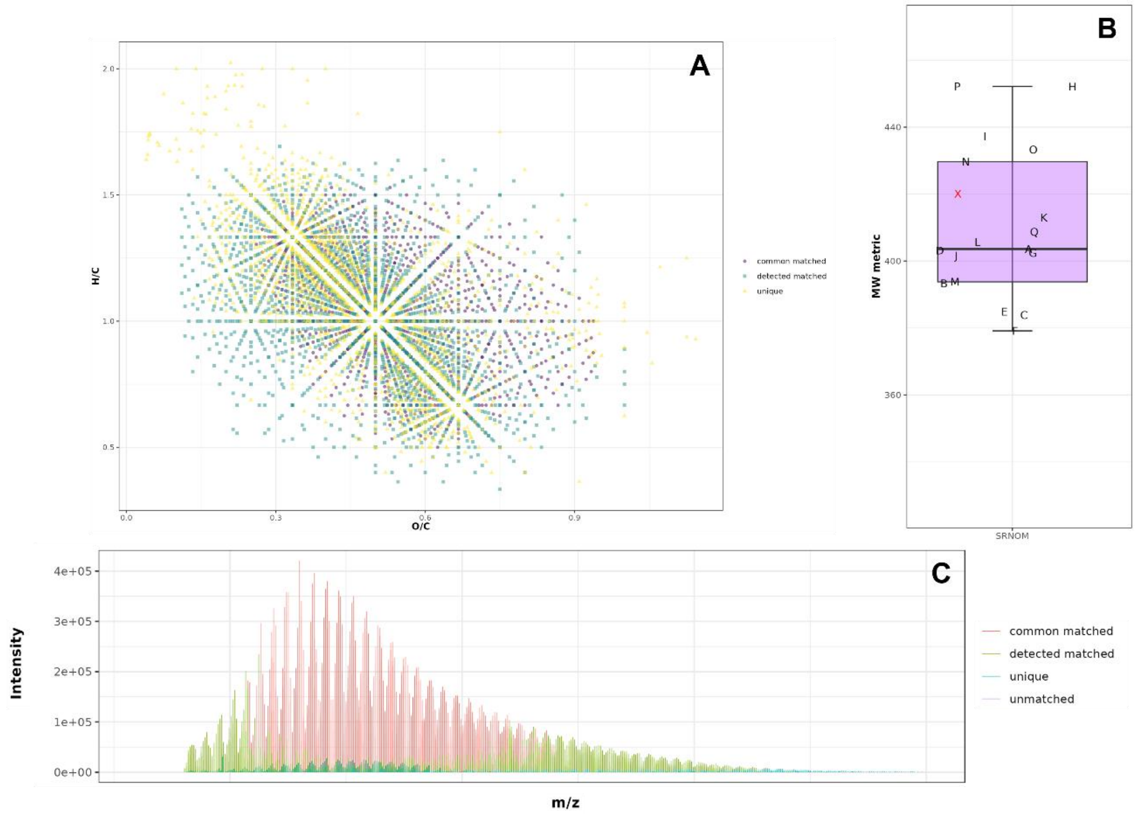

Figure 1. Comparison of SRNOM molecular formulas matched and not matched to the InterLabStudy dataset. (A) Van Krevelen diagram (H/C vs O/C) showing the distribution of matched and non-matched formulas. (B) Molecular mass distribution. (C) Signal intensity distribution, highlighting that non-matched formulas are enriched at lower intensities.

References

(1) Zhang, H.; Zhang, Y.; Shi, Q.; Zheng, H.; Yang, M. Characterization of Unknown Brominated Disinfection Byproducts during Chlorination Using Ultrahigh Resolution Mass Spectrometry. *Environ. Sci. Technol.* **2014**, *48* (6), 3112–3119. https://doi.org/10.1021/es4057399.

(2) Sanchís, J.; Petrović, M.; Farré, M. J. Emission of (Chlorinated) Reclaimed Water into a Mediterranean River and Its Related Effects to the Dissolved Organic Matter Fingerprint. *Science of the Total Environment* **2021**, *760*. https://doi.org/10.1016/j.scitotenv.2020.143881.

(3) Sanchís, J.; Jaén-Gil, A.; Gago-Ferrero, P.; Munthali, E.; Farré, M. J. Characterization of Organic Matter by HRMS in Surface Waters: Effects of Chlorination on Molecular Fingerprints and Correlation with DBP Formation Potential. *Water Res.* **2020**, *176*. https://doi.org/10.1016/j.watres.2020.115743.

(4) Nguyen, P. Y.; Carvalho, G.; Reis, M. A. M.; Oehmen, A. A Review of the Biotransformations of Priority Pharmaceuticals in Biological Wastewater Treatment Processes. *Water Res.* **2021**, *188*. https://doi.org/10.1016/j.watres.2020.116446.

(5) Kristiana, I.; Lethorn, A.; Joll, C.; Heitz, A. To Add or Not to Add: The Use of Quenching Agents for the Analysis of Disinfection by-Products in Water Samples. *Water Res.* **2014**, *59*, 90–98. https://doi.org/10.1016/j.watres.2014.04.006.

(6) He, H.; Sun, N.; Li, L.; Ai, J.; Zhou, H.; Yang, X.; Yang, X.; Wang, D.; Zhang, W. Effects of Dissolved Organic Matter Removal and Molecular Transformation in Different Water Treatment Processes on Formation of Disinfection Byproducts. *Water Res.* **2023**, *245*. https://doi.org/10.1016/j.watres.2023.120626.

(7) Wang, X.; Zhang, H.; Zhang, Y.; Shi, Q.; Wang, J.; Yu, J.; Yang, M. New Insights into Trihalomethane and Haloacetic Acid Formation Potentials: Correlation with the Molecular Composition of Natural Organic Matter in Source Water. *Environ. Sci. Technol.* **2017**, *51* (4), 2015–2021. https://doi.org/10.1021/acs.est.6b04817.

(8) Chu, W. H.; Gao, N. Y.; Deng, Y.; Krasner, S. W. Precursors of Dichloroacetamide, an Emerging Nitrogenous DBP Formed during Chlorination or Chloramination. *Environ. Sci. Technol.* **2010**, *44* (10), 3908–3912. https://doi.org/10.1021/es100397x.

(9) Farré, M. J.; Jaén-Gil, A.; Hawkes, J.; Petrovic, M.; Catalán, N. Orbitrap Molecular Fingerprint of Dissolved Organic Matter in Natural Waters and Its Relationship with NDMA Formation Potential. *Science of the Total Environment* **2019**, *670*, 1019–1027. https://doi.org/10.1016/j.scitotenv.2019.03.280.

(10) Postigo, C.; Andersson, A.; Harir, M.; Bastviken, D.; Gonsior, M.; Schmitt-Kopplin, P.; Gago-Ferrero, P.; Ahrens, L.; Ahrens, L.; Wiberg, K. Unraveling the Chemodiversity of Halogenated Disinfection By-Products Formed during Drinking Water Treatment Using Target and Non-Target Screening Tools. *J. Hazard. Mater.* **2021**, *401*. https://doi.org/10.1016/j.jhazmat.2020.123681.

(11) Zhang, Z.; Hu, S.; Sun, G.; Wang, W. Target Analysis, Occurrence and Cytotoxicity of Halogenated Polyhydroxyphenols as Emerging Disinfection Byproducts in Drinking Water. *Water Res.* **2024**, *248*. https://doi.org/10.1016/j.watres.2023.120883.

(12) Hua, G.; Reckhow, D. A. Comparison of Disinfection Byproduct Formation from Chlorine and Alternative Disinfectants. *Water Res.* **2007**, *41* (8), 1667–1678. https://doi.org/10.1016/j.watres.2007.01.032.

(13) Zhang, D.; Bond, T.; Li, M.; Dong, S.; Pan, Y.; Du, E.; Xiao, R.; Chu, W. Ozonation Treatment Increases Chlorophenylacetonitrile Formation in Downstream Chlorination or Chloramination. *Environ. Sci. Technol.* **2021**, *55* (6), 3747–3755. https://doi.org/10.1021/acs.est.0c07853.

(14) Duirk, S. E.; Lindell, C.; Cornelison, C. C.; Kormos, J.; Ternes, T. A.; Attene-Ramos, M.; Osiol, J.; Wagner, E. D.; Plewa, M. J.; Richardson, S. D. Formation of Toxic Iodinated Disinfection By-Products from Compounds Used in Medical Imaging. *Environ. Sci. Technol.* **2011**, *45* (16), 6845–6854. https://doi.org/10.1021/es200983f.

(15) Milstead, R. P.; Remucal, C. K. Molecular-Level Insights into the Formation of Traditional and Novel Halogenated Disinfection Byproducts. *ACS ES and T Water* **2021**, *1* (8), 1966–1974. https://doi.org/10.1021/acsestwater.1c00161.

(16) Maizel, A. C.; Li, J.; Remucal, C. K. Relationships between Dissolved Organic Matter Composition and Photochemistry in Lakes of Diverse Trophic Status. *Environ. Sci. Technol.* **2017**, *51* (17), 9624–9632. https://doi.org/10.1021/acs.est.7b01270.

(17) Munthali, E.; Marcé, R.; Farré, M. J. Drivers of Variability in Disinfection By-Product Formation Potential in a Chain of Thermally Stratified Drinking Water Reservoirs. *Environ. Sci. (Camb).* **2022**, *8* (5), 968–980. https://doi.org/10.1039/d1ew00788b.

(18) Lavonen, E. E.; Gonsior, M.; Tranvik, L. J.; Schmitt-Kopplin, P.; Köhler, S. J. Selective Chlorination of Natural Organic Matter: Identification of Previously Unknown Disinfection Byproducts. *Environ. Sci. Technol.* **2013**, *47* (5), 2264–2271. https://doi.org/10.1021/es304669p.

(19) Li, Y.; Harir, M.; Uhl, J.; Kanawati, B.; Lucio, M.; Smirnov, K. S.; Koch, B. P.; Schmitt-Kopplin, P.; Hertkorn, N. How Representative Are Dissolved Organic Matter (DOM) Extracts? A Comprehensive Study of Sorbent Selectivity for DOM Isolation. *Water Res.* **2017**, *116*, 316–323. https://doi.org/10.1016/j.watres.2017.03.038.

(20) Hawkes, J. A.; Dittmar, T.; Patriarca, C.; Tranvik, L.; Bergquist, J. Evaluation of the Orbitrap Mass Spectrometer for the Molecular Fingerprinting Analysis of Natural Dissolved Organic Matter. *Anal. Chem.* **2016**, *88* (15), 7698–7704. https://doi.org/10.1021/acs.analchem.6b01624.

(21) Patriarca, C.; Bergquist, J.; Sjöberg, P. J. R.; Tranvik, L.; Hawkes, J. A. Online HPLC-ESI-HRMS Method for the Analysis and Comparison of Different Dissolved Organic Matter Samples. *Environ. Sci. Technol.* **2018**, *52* (4), 2091–2099. https://doi.org/10.1021/acs.est.7b04508.

(22) Schum, S. K.; Brown, L. E.; Mazzoleni, L. R. MFAssignR: Molecular Formula Assignment Software for Ultrahigh Resolution Mass Spectrometry Analysis of Environmental Complex Mixtures. *Environ. Res.* **2020**, *191*. https://doi.org/10.1016/j.envres.2020.110114.

(23) Hawkes, J. A.; D'Andrilli, J.; Agar, J. N.; Barrow, M. P.; Berg, S. M.; Catalán, N.; Chen, H.; Chu, R. K.; Cole, R. B.; Dittmar, T.; Gavard, R.; Gleixner, G.; Hatcher, P. G.; He, C.; Hess, N. J.; Hutchins, R. H. S.; Ijaz, A.; Jones, H. E.; Kew, W.; Khaksari, M.; Palacio Lozano, D. C.; Lv, J.; Mazzoleni, L. R.; Noriega-Ortega, B. E.; Osterholz, H.; Radoman, N.; Remucal, C. K.; Schmitt, N. D.; Schum, S. K.; Shi, Q.; Simon, C.; Singer, G.; Sleighter, R. L.; Stubbins, A.; Thomas, M. J.; Tolic, N.; Zhang, S.; Zito, P.; Podgorski, D. C. An International Laboratory Comparison of Dissolved Organic Matter Composition by High Resolution Mass Spectrometry: Are We Getting the Same Answer? *Limnol. Oceanogr. Methods* **2020**, *18* (6), 235–258. https://doi.org/10.1002/lom3.10364.

(24) Schymanski, E. L.; Jeon, J.; Gulde, R.; Fenner, K.; Ruff, M.; Singer, H. P.; Hollender, J. Identifying Small Molecules via High Resolution Mass Spectrometry: Communicating Confidence. *Environmental Science and Technology*. February 18, 2014, pp 2097–2098. https://doi.org/10.1021/es5002105.

(25) Kellerman, A. M.; Dittmar, T.; Kothawala, D. N.; Tranvik, L. J. Chemodiversity of Dissolved Organic Matter in Lakes Driven by Climate and Hydrology. *Nat. Commun.* **2014**, *5*. https://doi.org/10.1038/ncomms4804.

(26) https://kairos.warwick.ac.uk/InterLabStudy/

Supplementary figures and tables

Table S1.

Drinking water treatment plant conditions during the samplings.

Campaign	Sampling	Date	Flow (m^3/s)	%Desalinated water in the tanks	Discharge of Chlorine dioxide (Preoxidation-Clarified samples) (mg/L)	Discharge of Chlorine (Disinfection process – Produced water samples) (mg/L)
1	1	17/04/2023	2.87	13.6	1.00	0.80
1	2	24/04/2023	2.87	13.6	1.00	0.80
2	3	06/11/2023	3.38	9.7	0.45	1.65
2	4	20/11/2023	3.89	10.8	0.28	1.30
3	5	08/01/2024	2.19	14.6	0.25	1.10
3	6	29/01/2024	1.87	9.8	0.45	1.82

Table S2.

Limits of quantification of general parameters measured for DWTP samples. Units in mg/L or µS/cm

Cond	NO_2	NO_3	PO_4	Cl	SO_4	Br	F	ClO_2	ClO_3	I	Na	NH_4	TH	DOC	TN
0.2	0.003	0.002	0.008	0.0025	0.01	0.01	0.001	0.005	0.004	0.025	0.025	0.004	0.166	0.05	0.05

Table S3.

Concentration ranges for the general parameters for all samples.

Sample	pH	Cond	NO2	NO3	PO4	Cl	SO4	Br	F	ClO2	ClO3	I	Na	NH4	TH	DOC	TN
Raw	7.71-8.24	460-520	LOQ-0.004	0.57-1.26	LOQ-0.01	34.8-42.8	19.9-22.9	LOQ	0.11-0.13	LOQ	LOQ	LOQ	29.0-35.1	LOQ	156-182	2.13-3.61	0.62-1.57
Clarified	7.30-7.97	489-535	LOQ	0.59-1.27	LOQ-0.16	42.9-49.7	19.8-22.4	LOQ	0.10-0.11	0.15-0.27	0.02-0.29	LOQ	31.3-37.0	LOQ-0.004	157-181	1.93-3.21	1.07-1.59
Post AC	7.05-8.12	502-539	LOQ-0.065	0.57-1.28	LOQ	44.4-51.3	19.3-22.7	LOQ	0.01-0.12	LOQ-0.04	0.04-0.21	LOQ	33.3-38.5	LOQ-0.02	156-181	1.16-2.71	1.04-1.49
Produced water	7.70-8.10	558-654	LOQ	0.38-0.96	LOQ	69.1-98.3	13.9-26.1	LOQ-0.11	0.07-0.09	LOQ-0.07	0.04-0.16	LOQ	49.1-62.2	LOQ-0.01	130-154	0.89-1.46	0.79-1.12
Distribution 24h	7.10-8.11	494-551	LOQ	0.53-1.16	LOQ	46.5-55.8	17.9-21.5	LOQ-0.06	0.10-0.10	LOQ-0.01	0.05-0.22	LOQ	31.4-46.6	LOQ	158-179	1.32-1.97	0.95-1.44
Distribution 48h	7.33-8.17	502-552	LOQ	0.48-1.14	LOQ	48.9-60.3	15.9-21.3	LOQ-0.20	0.09-0.10	LOQ-0.07	0.04-0.21	LOQ	35.5-41.9	LOQ-0.004	151-178	1.31-9.99	0.90-1.38
Distribution 72h	7.18-8.05	497-555	LOQ	0.50-1.14	LOQ	50.3-59.2	16.4-21.4	LOQ-0.11	0.09-0.10	LOQ-0.06	0.04-0.21	LOQ	34.9-42.2	LOQ-0.01	154-177	1.27-1.61	0.88-1.35

Note: n equal to 6.

Table S4.

% Recovery of DOM extraction.

Sample	Sampling 1	Sampling 2	Sampling 3	Sampling 4	Sampling 5	Sampling 6
Raw	72.26	50.00	82.78	45.18	61.75	54.83
Clarified	74.61	60.33	83.73	71.33	65.50	49.33
Post AC	36.49	45.86	30.66	48.35	42.30	43.38
Produced water	47.60	54.09	49.23	57.45	45.98	50.73
Distribution 24h	65.13	38.34	74.20	32.83	56.05	43.85
Distribution 48h	52.90	37.61	64.98	7.41	40.83	39.60
Distribution 72h	47.01	56.88	52.13	74.43	41.90	39.33
Raw FP	51.75	50.78	-	-	52.00	49.05
Clarified FP	40.32	38.45	-	-	44.20	33.43
Post AC FP	45.87	51.23	-	-	36.38	58.73
Produced water FP	50.22	47.96	-	-	48.60	49.43

Table S5.

First 10 molecular formulae presented in at least in 4 of 6 samples and employed for all data analysis and representations

Raw			Clarified			Post AC			Produced water			Distribution 24h			Distribution 48h			Distribution 72h		
F	G	C	F	G	C	F	G	C	F	G	C	F	G	C	F	G	C	F	G	C
C9H9O2Br	O2Br	CHOBr	C10H10N2O3	N2O3	CHNO	C10H10N2O3	N2O3	CHNO	C10H10N2O3	N2O3	CHNO	C10H10N2O3	N2O3	CHNO	C10H10N2O3	N2O3	CHNO	C10H10N2O3	N2O3	CHNO
C17H20N2O10	N2O10	N2O10	C10H10N2O4	N2O4	CHNO	C10H10N2O4	N2O4	CHNO	C10H10N2O4	N2O4	CHNO	C10H10N2O4	N2O4	CHNO	C10H10N2O4	N2O4	CHNO	C10H10N2O4	N2O4	CHNO
C15H12N2O10	N2O10	CHNO	C10H10N2O5	N2O5	CHNO	C10H10N2O5	N2O5	CHNO	C10H10N2O5	N2O5	CHNO	C10H10N2O5	N2O5	CHNO	C10H10N2O5	N2O5	CHNO	C10H10N2O5	N2O5	CHNO
C22H26N2O10	N2O10	CHNO	C10H10N2O6	N2O6	CHNO	C10H10N2O6	N2O6	CHNO	C10H10N2O6	N2O6	CHNO	C10H10N2O6	N2O6	CHNO	C10H10N2O6	N2O6	CHNO	C10H10N2O6	N2O6	CHNO
C15H14N2O10	N2O10	CHNO	C10H10O3S	O3S	CHOS	C10H10O3S	O3S	CHOS	C10H10O3S	O3S	CHOS	C10H10O3S	O3S	CHOS	C10H10O3S	O3S	CHOS	C10H10O3S	O3S	CHOS
C16H10N2O10	N2O10	CHNO	C10H10O4S	O4S	CHOS	C10H10O4S	O4S	CHOS	C10H10O4S	O4S	CHOS	C10H10O4S	O4S	CHOS	C10H10O4S	O4S	CHOS	C10H10O4S	O4S	CHOS
C16H12N2O10	N2O10	CHNO	C10H10O5	O5	CHO	C10H10O5	O5	CHO	C10H10O5	O5	CHO	C10H10O5	O5	CHO	C10H10O5	O5	CHO	C10H10O5	O5	CHO
C16H14N2O10	N2O10	CHNO	C10H10O5S	O5S	CHOS	C10H10O5S	O5S	CHOS	C10H10O5S	O5S	CHOS	C10H10O5S	O5S	CHOS	C10H10O5S	O5S	CHOS	C10H10O5S	O5S	CHOS
C16H16N2O10	N2O10	CHNO	C10H10O6	O6	CHO	C10H10O6	O6	CHO	C10H10O6	O6	CHO	C10H10O6	O6	CHO	C10H10O6	O6	CHO	C10H10O6	O6	CHO
C17H12N2O10	N2O10	CHNO	C10H10O6S	O6S	CHOS	C10H10O6S	O6S	CHOS	C10H10O6S	O6S	CHOS	C10H10O6S	O6S	CHOS	C10H10O6S	O6S	CHOS	C10H10O6S	O6S	CHOS

Note. F: Formulae; G: Group; C: Class. The rest of the dataset is provided under request.

Table S6.

Weighted averages of DBE_w, O/C_w, H/C_w and $DBE\text{-}O_w$ per sample

Sample	DBE_w	O/C_w	H/C_w	$DBE\text{-}O_w$
Raw	7.77±0.08	$0.3\pm6*10^{-5}$	$1.0\pm4*10^{-5}$	$4.0\pm7*10^{-3}$
Clarified	7.11±0.07	$0.3\pm4*10^{-5}$	$1.0\pm5*10^{-6}$	$4.0\pm8*10^{-3}$
Post AC	7.09±0.05	$0.3\pm8*10^{-5}$	$1.0\pm4*10^{-6}$	$4.0\pm5*10^{-3}$
Produced water	7.06±0.03	$0.3\pm7*10^{-5}$	$1.0\pm2*10^{-6}$	$4.0\pm6*10^{-3}$
Distribution 24h	7.04±0.02	$0.3\pm2*10^{-5}$	$1.0\pm3*10^{-6}$	$4.0\pm3*10^{-3}$
Distribution 48h	7.05±0.06	$0.3\pm3*10^{-5}$	$1.0\pm8*10^{-6}$	$4.0\pm2*10^{-3}$
Distribution 72h	7.02±0.04	$0.3\pm5*10^{-4}$	$1.0\pm7*10^{-6}$	$4.0\pm7*10^{-3}$

Note. Parameters calculated following the method of Maizel et al.

Table S7.

Criteria for formula assignment according to their regions in the Van Krevelen diagram.

Class	H/C ratio	O/C ratio	AImod value
Aliphatic-like	≥1.5	-	-
Aromatics-like	-	-	≥0.67
Condensed aromatics-like	-	-	0.5 < AImod < 0.67
High O unsaturated-like	<1.5	≥0.5	<0.5
Low O unsaturated-like	<1.5	<0.5	<0.67

Note. Hawkes, J. A., d'Andrilli, J., Agar, J. N., Barrow, M. P., Berg, S. M., Catalán, N., ... & Podgorski, D. C. (2020). An international laboratory comparison of dissolved organic matter composition by high resolution mass spectrometry: Are we getting the same answer?. Limnology and Oceanography: Methods, 18(6), 235-258.

Table S8.

Compound number ranges for each DWTP sample points.

Sample Point	Aliphatics-like	Aromatics-like	Condensed Aromatics-like	High O Unsaturated-like	Low O Unsaturated-like	Total
Raw	752-783	303-378	152-195	1329-1436	1612-1805	4175-4483
Clarified	765-839	175-196	93-137	1148-1308	1548-1747	3853-4087
Post AC	768-833	137-161	67-102	1186-1288	1484-1726	3656-4031
Produced Water	763-842	145-184	73-120	1150-1420	1445-1661	3608-4227
Distribution 24h	743-834	140-162	67-121	1133-1333	1421-1631	3605-3989
Distribution 48h	723-855	136-154	82-109	1127-1230	1409-1597	3577-3815
Distribution 72h	708-835	130-164	72-129	1058-1242	1364-1545	3522-3827

Note: n equal to 6.

Table S9.

Disinfection byproducts concentrations (in µg/L) of the formation potential test

DBP	Raw FP 1	Raw FP 2	Raw FP 3	Raw FP 4	Raw FP 5	Raw FP 6
TCM	43.23	47.92	45.7	38.46	41.23	35.70
BDCM	7.30	13.79	10.55	12.13	12.34	11.91
DBCM	1.64	2.31	1.98	2.94	2.81	3.06
TBM	0.15	0.15	0.15	0.16	0.15	0.16
TTHMs	52.32	64.17	58.25	35.84	56.54	15.13
DCAN	4.36	5.86	5.11	6.27	6.89	5.65
BCAN	1.92	1.89	1.90	2.12	2.30	1.93
DBAN	0.92	1.06	0.99	0.19	0.29	0.08
HANs	7.19	8.81	8.00	8.57	9.49	7.66
MBAA	1.00	0.43	0.71	0.21	0.03	0.39
DCAA	20.92	21.86	21.39	22.78	23.14	22.43
TCAA	35.49	35.70	35.59	30.54	27.94	33.14
BCAA	3.93	4.55	4.24	4.88	4.53	5.23
DBAA	0.48	0.61	0.55	0.89	0.83	0.95
HAAs	61.07	60.24	60.66	55.57	51.91	59.24

Table S10.

Number of highly correlated features and classified according to their region in the van Krevelen presented in each sample

Sample	Aliphatic	Aromatic	Condensed aromatic	High-Oxygen unsaturated	Low-Oxygen unsaturated	Total
Raw	38	3	0	18	38	97
Clarified	33	2	0	4	19	58
Post AC	35	1	0	6	19	61
Produced water	31	0	0	5	17	53
Distribution 24h	29	0	0	2	15	46
Distribution 48h	32	0	0	2	17	51
Distribution 72h	30	0	0	3	15	48

Figure S1. Chromatogram of SRNOM standard marking the DOM window of 4-9 minutes.

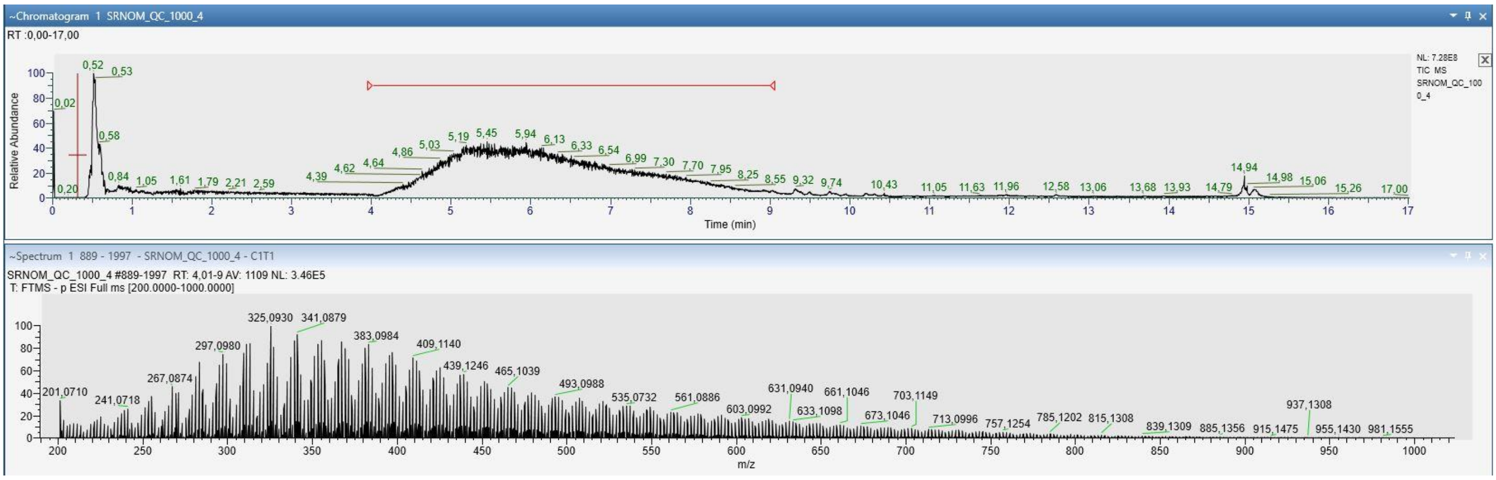

Figure S2. Distribution of deviations from the theoretical $^{37}Cl/^{35}Cl$ and $^{81}Br/^{79}Br$ isotopic ratios for assigned halogenated compounds. Dashed lines indicate zero deviation, and dotted lines represent the ±30% tolerance applied during isotopic pattern validation.

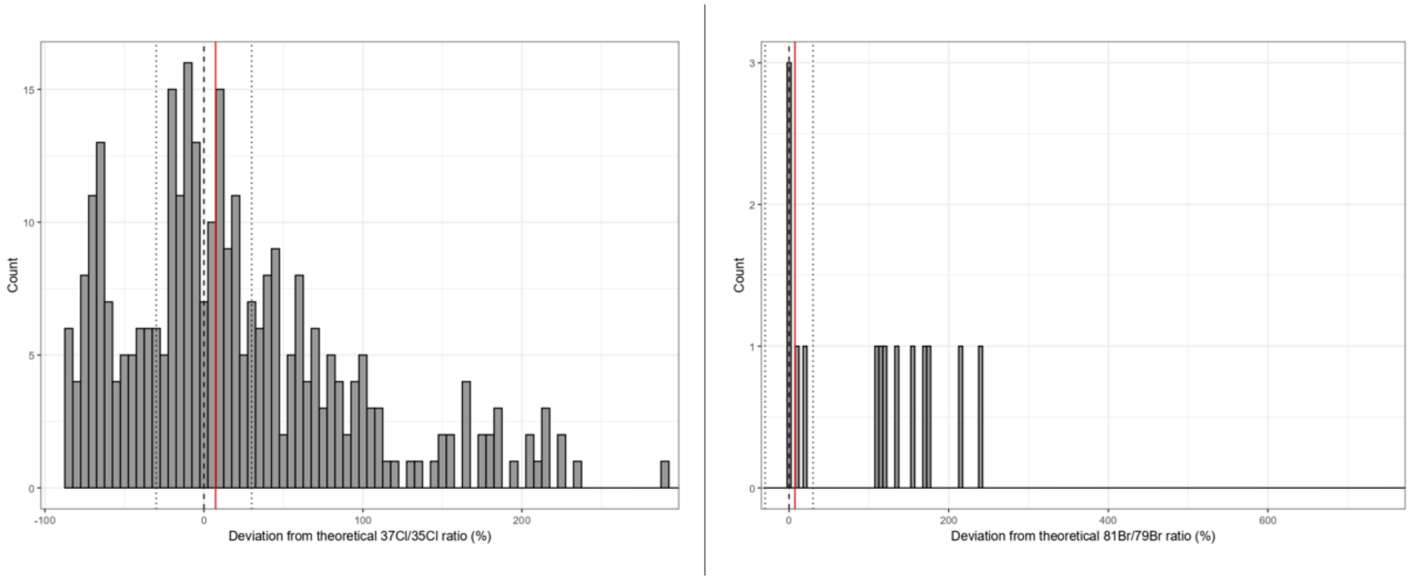

Figure S3. Distribution of mass errors (ppm) for chlorinated molecular formulas assigned from Orbitrap Exploris 120 HRMS data. The dashed line indicates zero mass error, and dotted lines represent the applied ±1 ppm tolerance window.

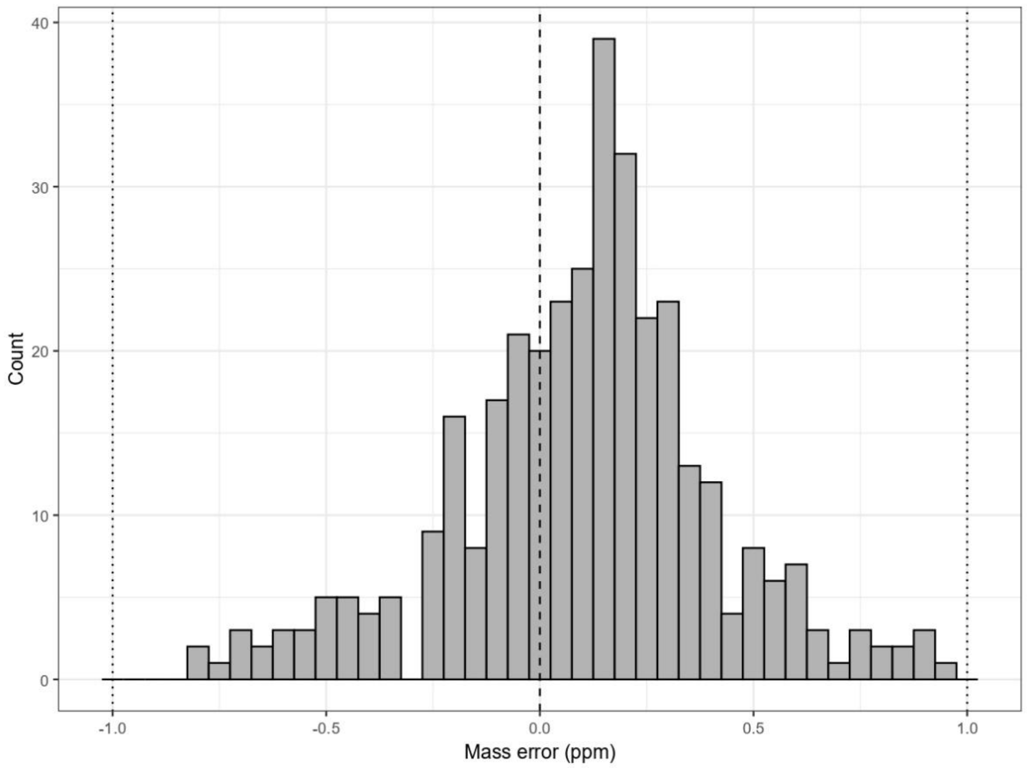

Figure S4. Van Krevelen diagram of SRNOM standard with common classification.

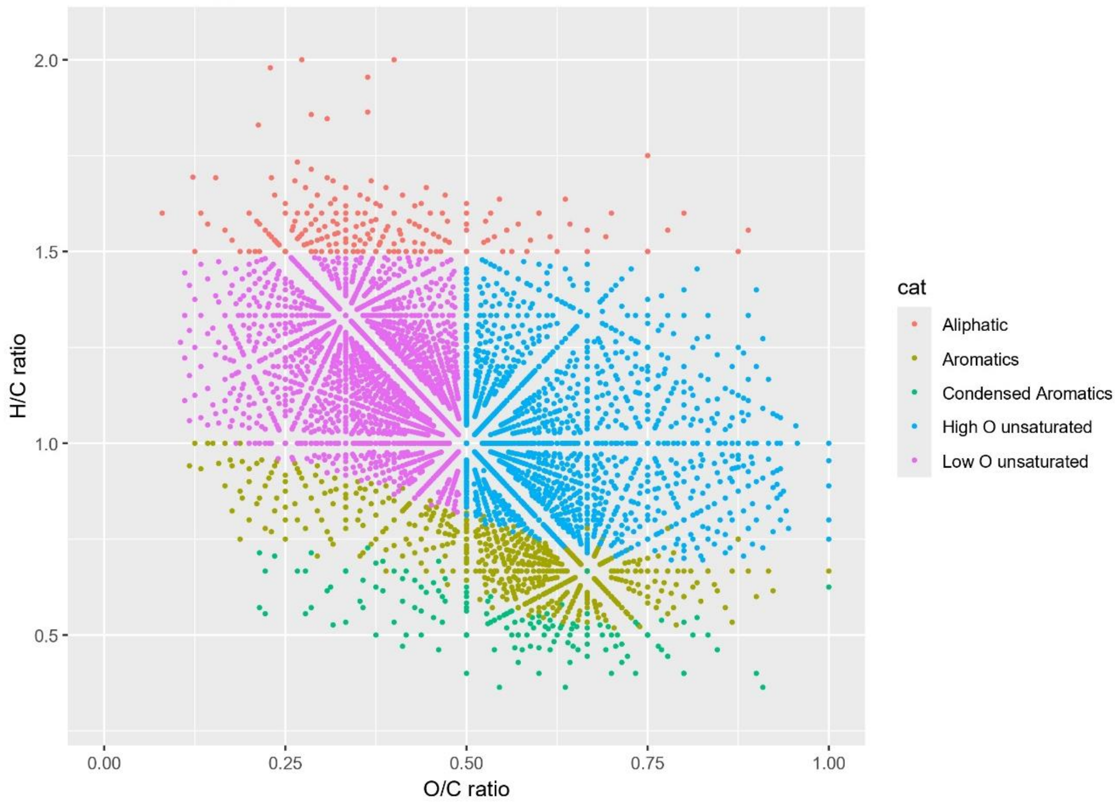

Figure S5. Distribution of the concentration (in μg/L) of DBPs among the DWTP.

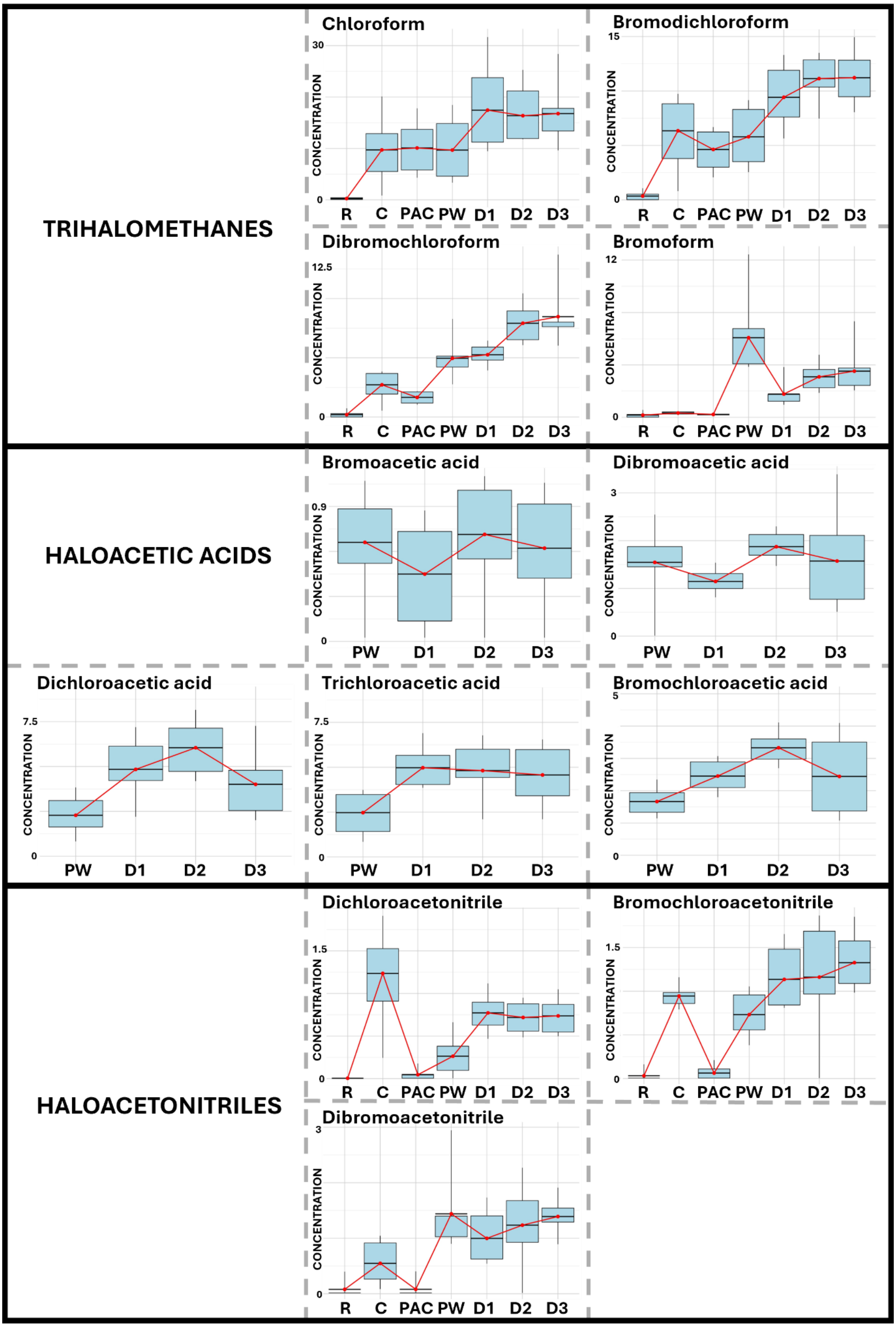

Figure S6. Percentages of known DBPs in DWTP water samples.

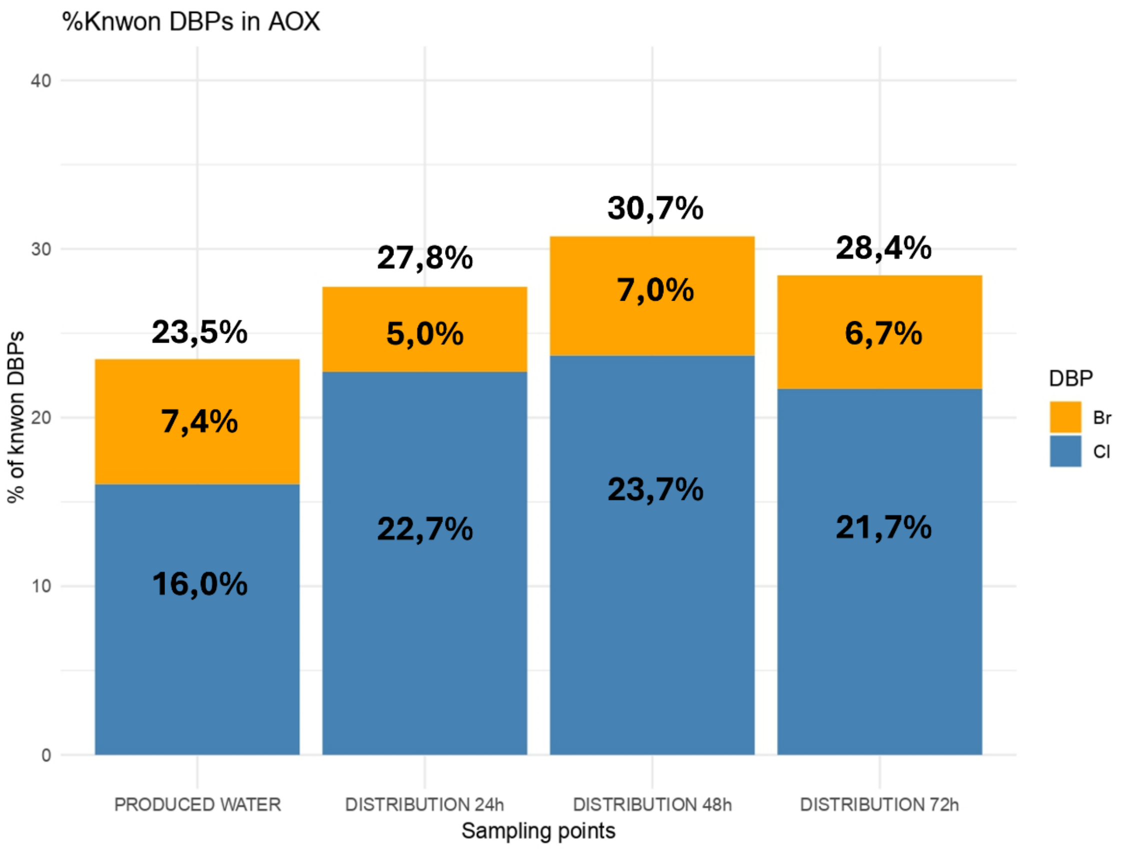

Figure S7. Example high-resolution mass spectrum showing the isotopic pattern of a representative chlorinated molecular formula detected in the samples. The monoisotopic peak (^{35}Cl) and the corresponding M+2 isotopic peak (^{37}Cl) are consistent with the expected chlorine isotopic signature.

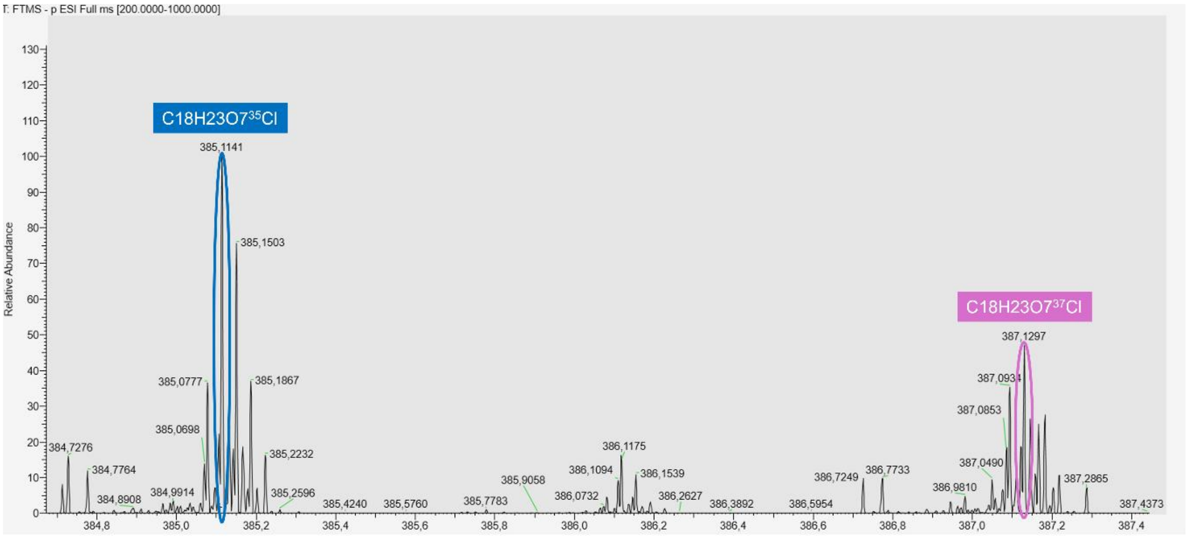

Figure S8. Intensity changes of each precursor throughout the treatment.

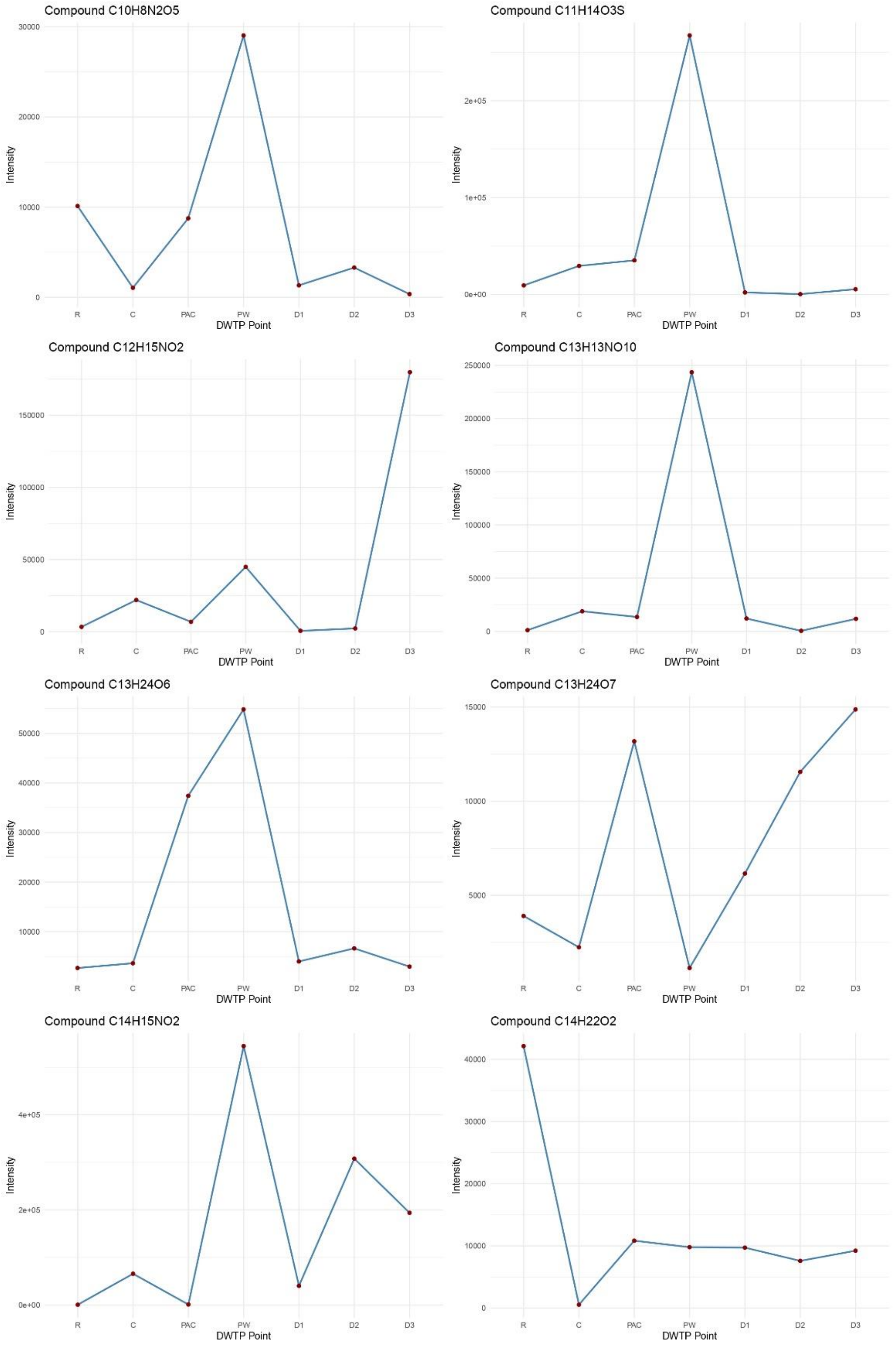

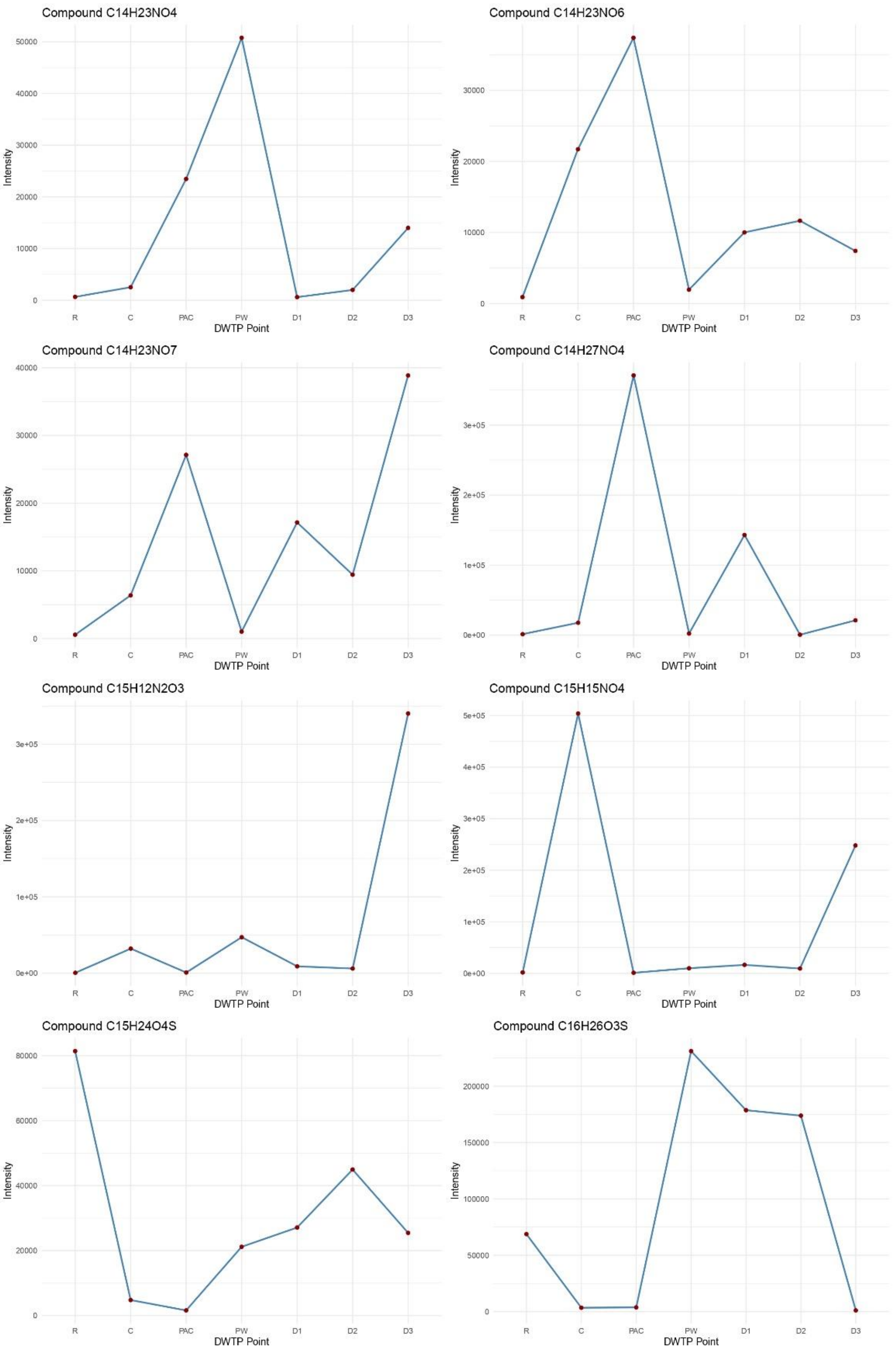
Compound C14H23NO4
Intensity
50000
40000
30000
20000
10000
0
R C PAC PW D1 D2 D3
DWTP Point
Compound C14H23NO6
Intensity
30000
20000
10000
0
R C PAC PW D1 D2 D3
DWTP Point
Compound C14H23NO7
Intensity
40000
30000
20000
10000
0
R C PAC PW D1 D2 D3
DWTP Point
Compound C14H27NO4
Intensity
3e+05
2e+05
1e+05
0e+00
R C PAC PW D1 D2 D3
DWTP Point
Compound C15H12N2O3
Intensity
3e+05
2e+05
1e+05
0e+00
R C PAC PW D1 D2 D3
DWTP Point
Compound C15H15NO4
Intensity
5e+05
4e+05
3e+05
2e+05
1e+05
0e+00
R C PAC PW D1 D2 D3
DWTP Point
Compound C15H24O4S
Intensity
80000
60000
40000
20000
0
R C PAC PW D1 D2 D3
DWTP Point
Compound C16H26O3S
Intensity
200000
150000
100000
50000
0
R C PAC PW D1 D2 D3
DWTP Point

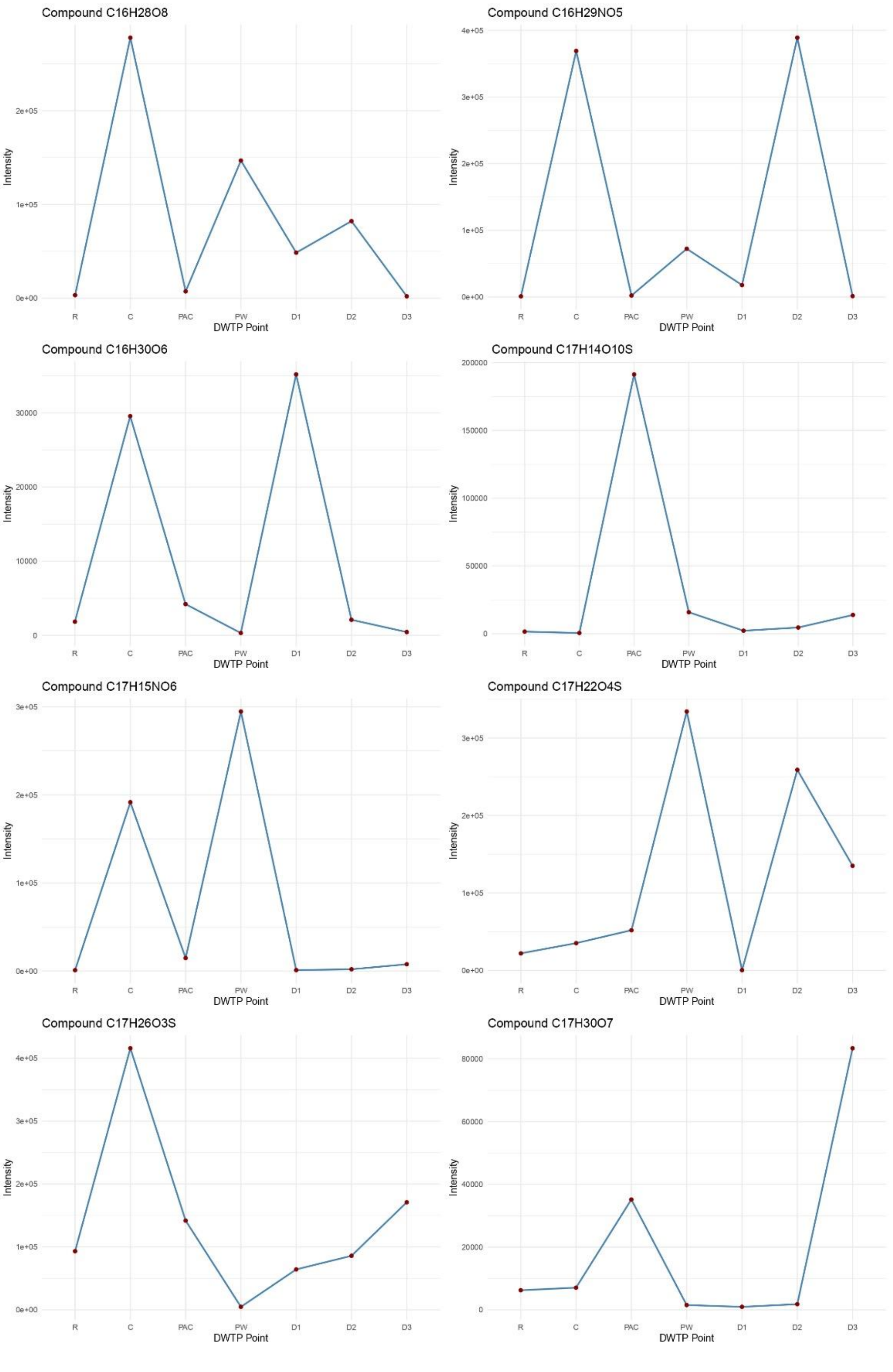
Compound C16H28O8
Compound C16H29NO5
Compound C16H30O6
Compound C17H14O10S
Compound C17H15NO6
Compound C17H22O4S
Compound C17H26O3S
Compound C17H30O7
Intensity
DWTP Point
R
C
PAC
PW
D1
D2
D3

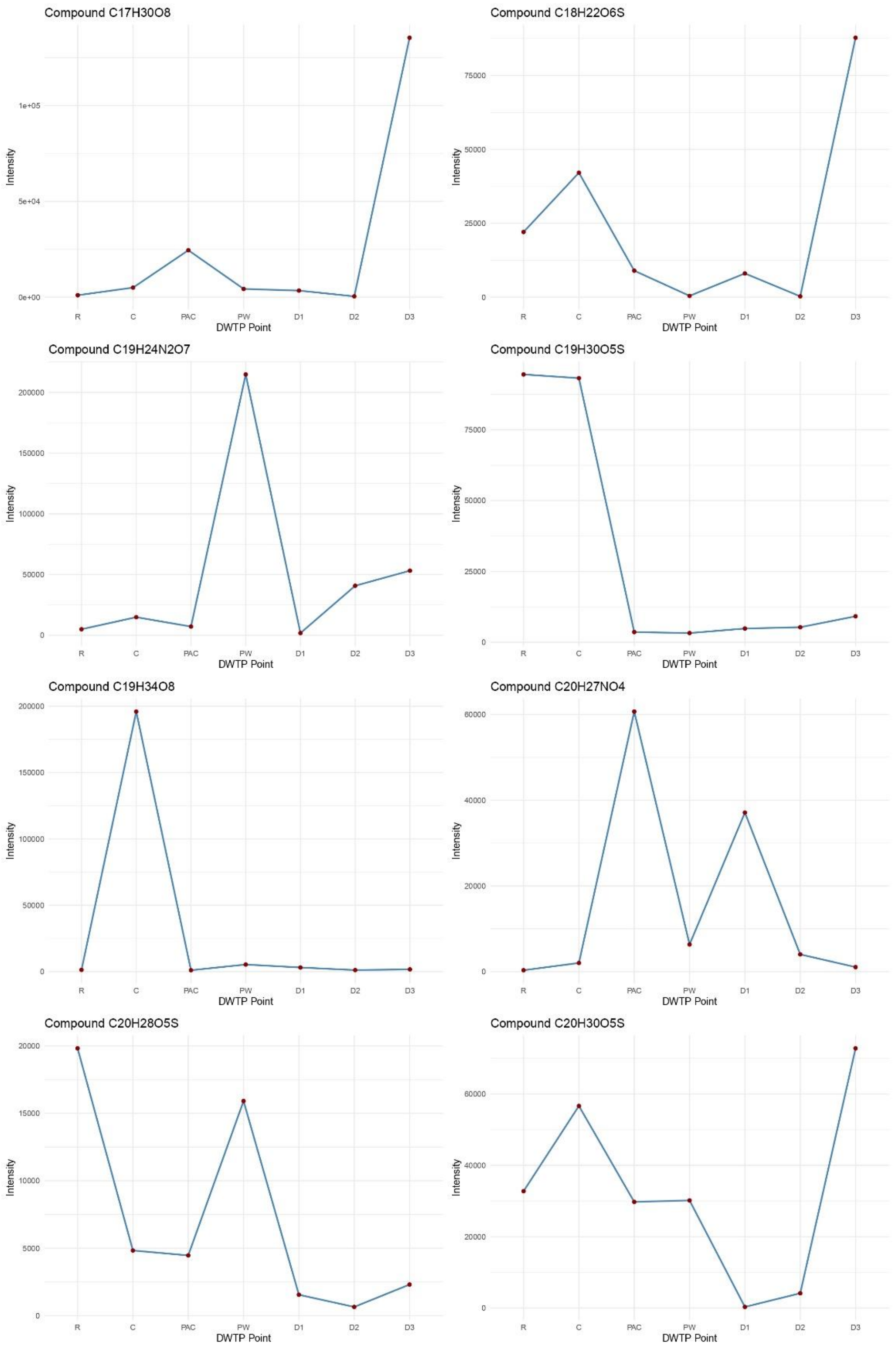

Compound C17H30O8
Compound C18H22O6S
Compound C19H24N2O7
Compound C19H30O5S
Compound C19H34O8
Compound C20H27NO4
Compound C20H28O5S
Compound C20H30O5S
Intensity
DWTP Point
R
C
PAC
PW
D1
D2
D3

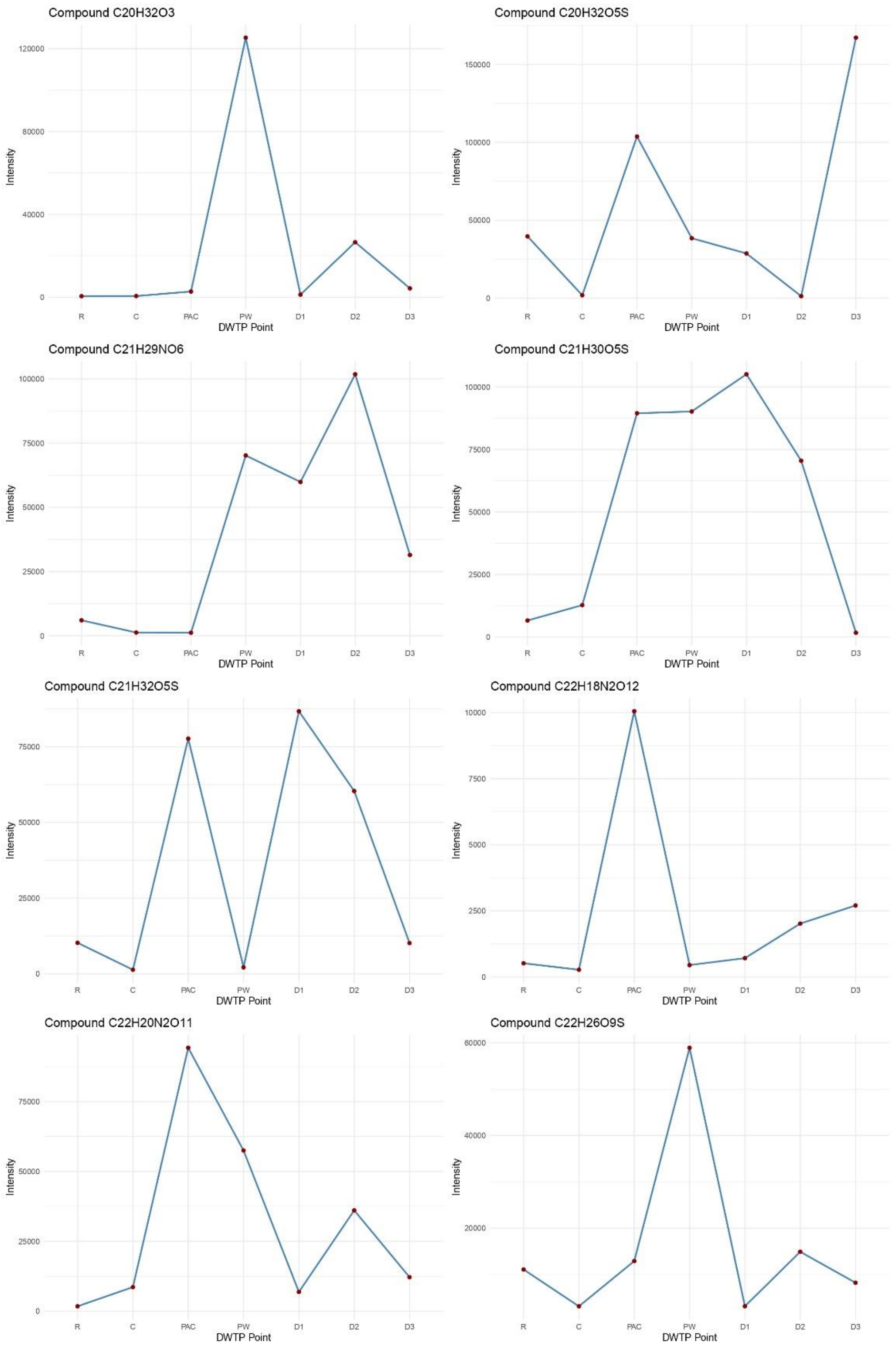
Compound C20H32O3
Intensity
0
40000
80000
120000
R
C
PAC
PW
D1
D2
D3
DWTP Point
Compound C20H32O5S
Intensity
0
50000
100000
150000
R
C
PAC
PW
D1
D2
D3
DWTP Point
Compound C21H29NO6
Intensity
0
25000
50000
75000
100000
R
C
PAC
PW
D1
D2
D3
DWTP Point
Compound C21H30O5S
Intensity
0
25000
50000
75000
100000
R
C
PAC
PW
D1
D2
D3
DWTP Point
Compound C21H32O5S
Intensity
0
25000
50000
75000
R
C
PAC
PW
D1
D2
D3
DWTP Point
Compound C22H18N2O12
Intensity
0
2500
5000
7500
10000
R
C
PAC
PW
D1
D2
D3
DWTP Point
Compound C22H20N2O11
Intensity
0
25000
50000
75000
R
C
PAC
PW
D1
D2
D3
DWTP Point
Compound C22H26O9S
Intensity
20000
40000
60000
R
C
PAC
PW
D1
D2
D3
DWTP Point

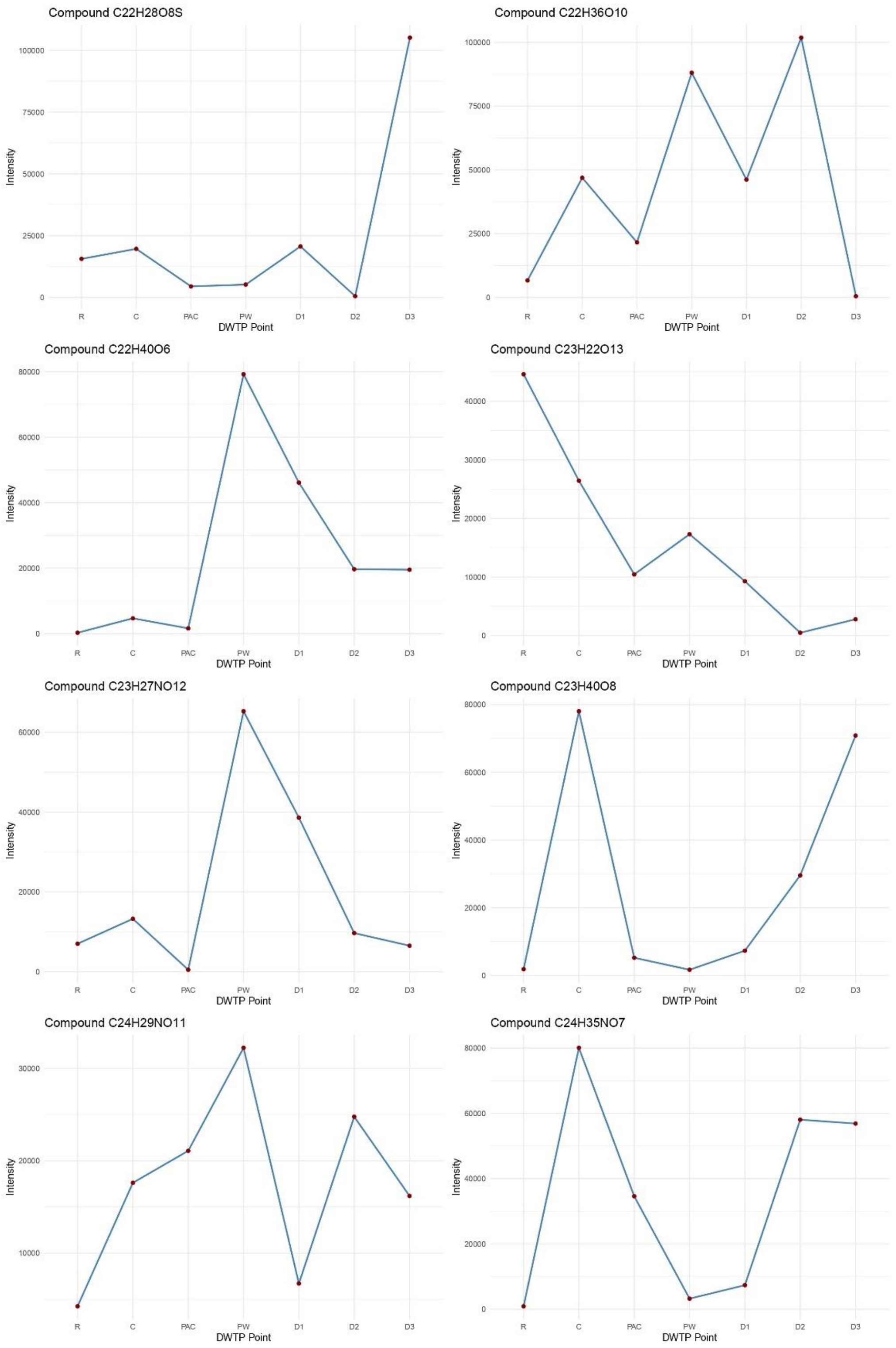
Compound C22H28O8S
Compound C22H36O10
Compound C22H40O6
Compound C23H22O13
Compound C23H27NO12
Compound C23H40O8
Compound C24H29NO11
Compound C24H35NO7
Intensity
DWTP Point
R
C
PAC
PW
D1
D2
D3

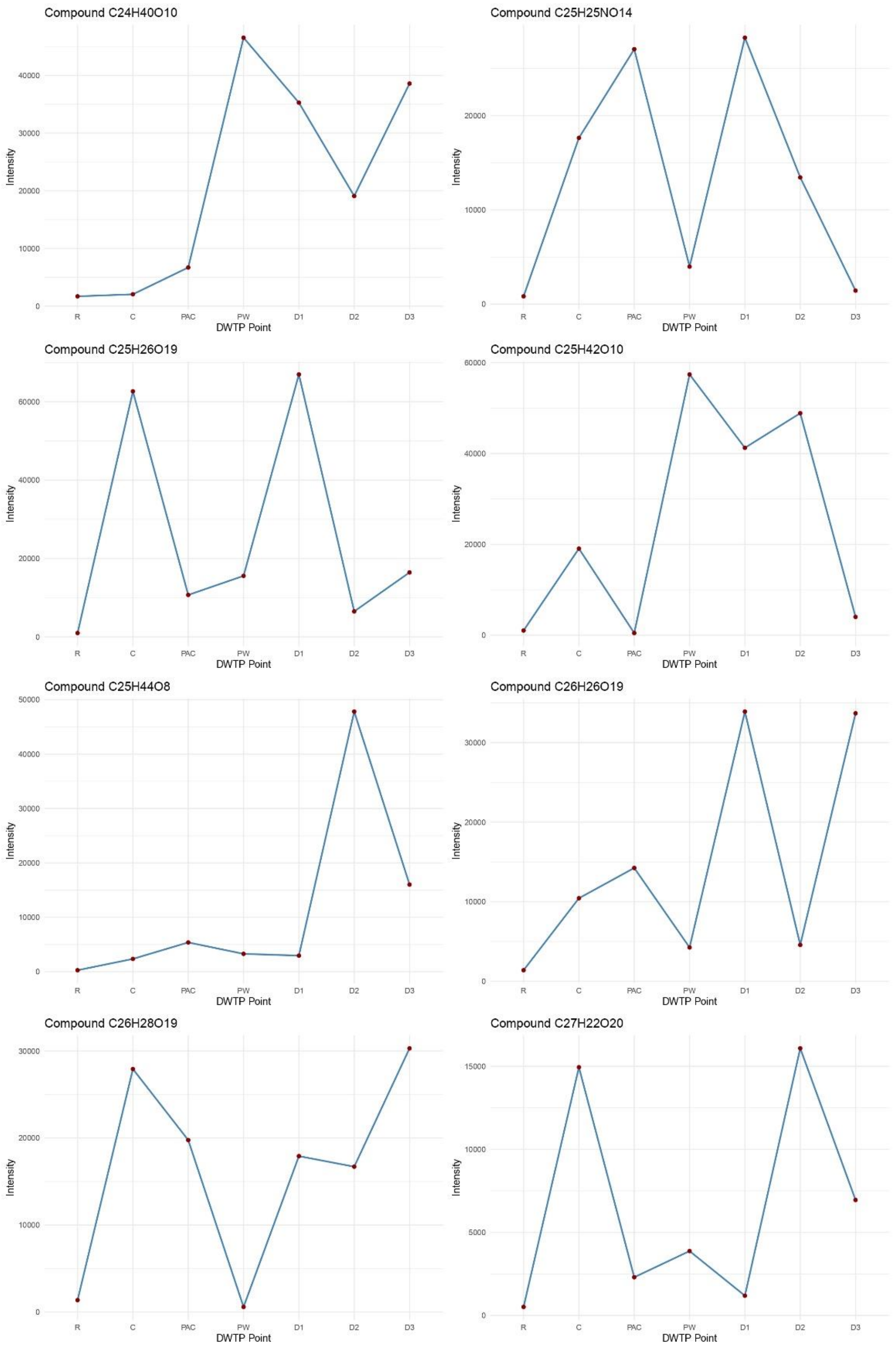
Compound C24H40O10
Intensity
0
10000
20000
30000
40000
R
C
PAC
PW
D1
D2
D3
DWTP Point
Compound C25H25NO14
Intensity
0
10000
20000
R
C
PAC
PW
D1
D2
D3
DWTP Point
Compound C25H26O19
Intensity
0
20000
40000
60000
R
C
PAC
PW
D1
D2
D3
DWTP Point
Compound C25H42O10
Intensity
0
20000
40000
60000
R
C
PAC
PW
D1
D2
D3
DWTP Point
Compound C25H44O8
Intensity
0
10000
20000
30000
40000
50000
R
C
PAC
PW
D1
D2
D3
DWTP Point
Compound C26H26O19
Intensity
0
10000
20000
30000
R
C
PAC
PW
D1
D2
D3
DWTP Point
Compound C26H28O19
Intensity
0
10000
20000
30000
R
C
PAC
PW
D1
D2
D3
DWTP Point
Compound C27H22O20
Intensity
0
5000
10000
15000
R
C
PAC
PW
D1
D2
D3
DWTP Point

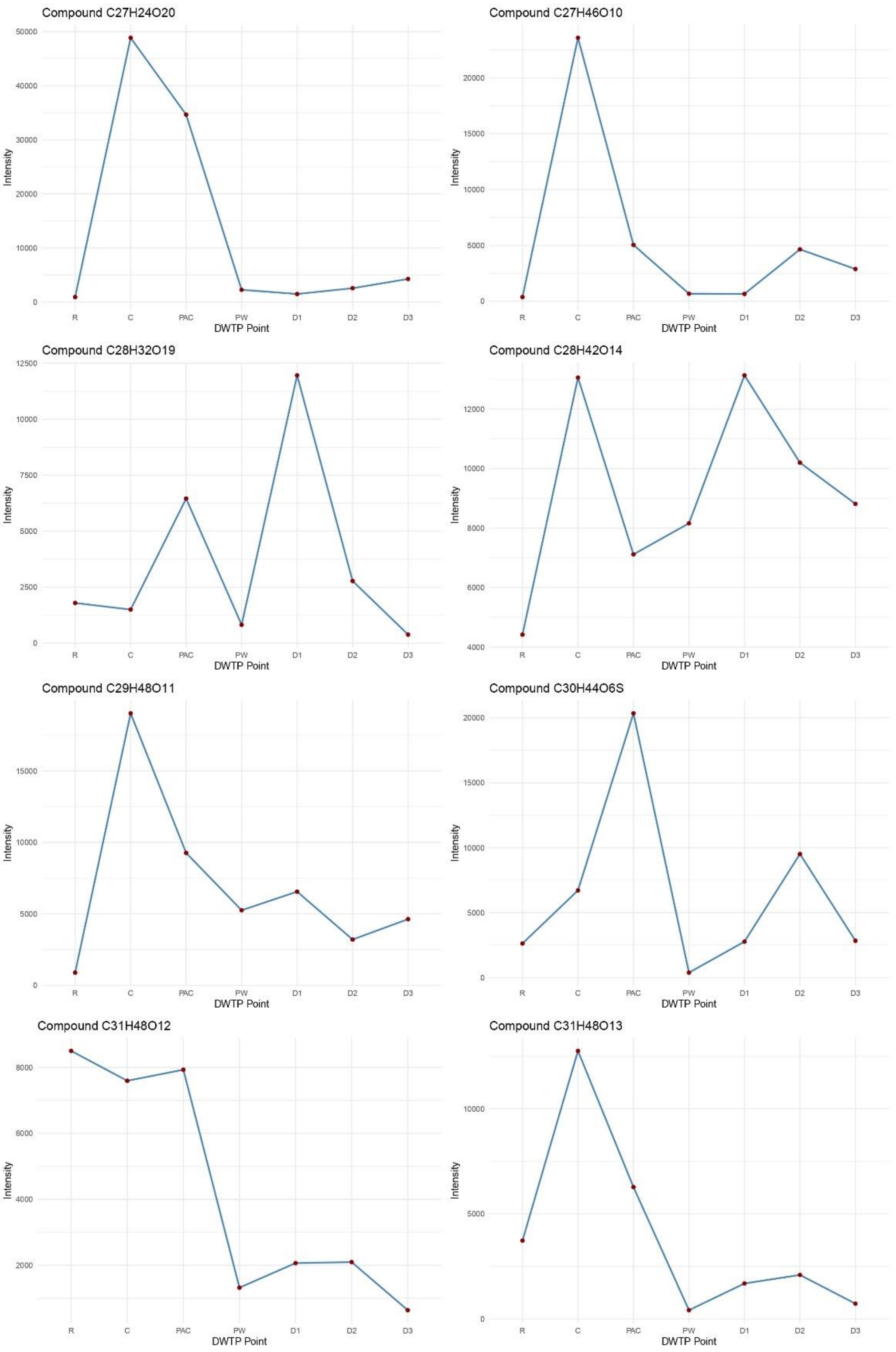
Compound C27H24O20
Compound C27H46O10
Compound C28H32O19
Compound C28H42O14
Compound C29H48O11
Compound C30H44O6S
Compound C31H48O12
Compound C31H48O13
Intensity
DWTP Point
R
C
PAC
PW
D1
D2
D3

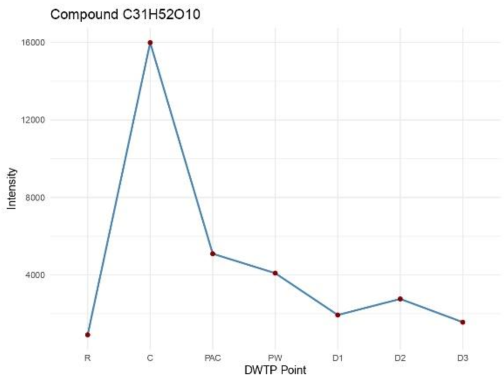
Compound C31H52O10
16000
12000
8000
4000
Intensity
R C PAC PW D1 D2 D3
DWTP Point

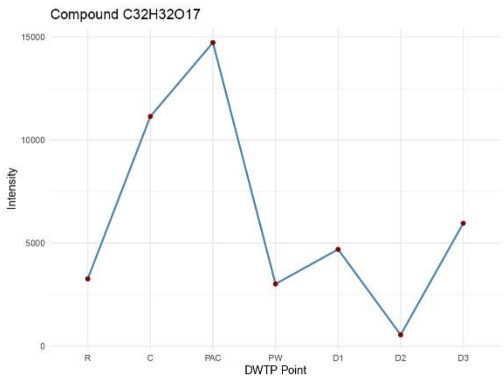
Compound C32H32O17
15000
10000
5000
0
Intensity
R C PAC PW D1 D2 D3
DWTP Point

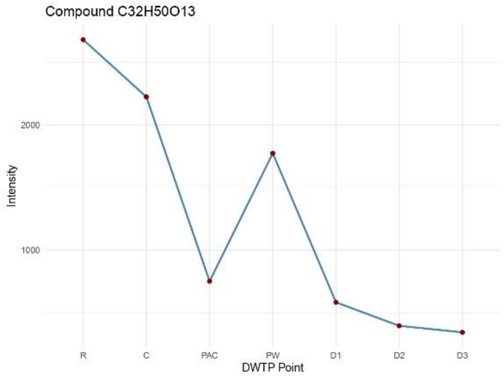
Compound C32H50O13
2000
1000
Intensity
R C PAC PW D1 D2 D3
DWTP Point

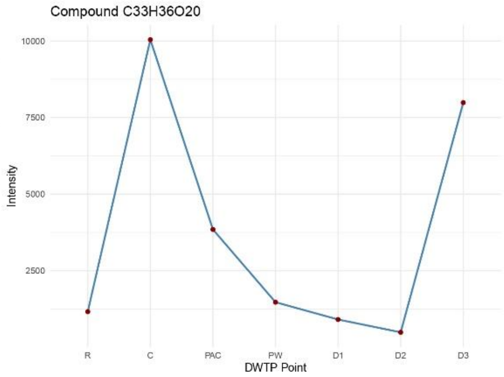
Compound C33H36O20
10000
7500
5000
2500
Intensity
R C PAC PW D1 D2 D3
DWTP Point

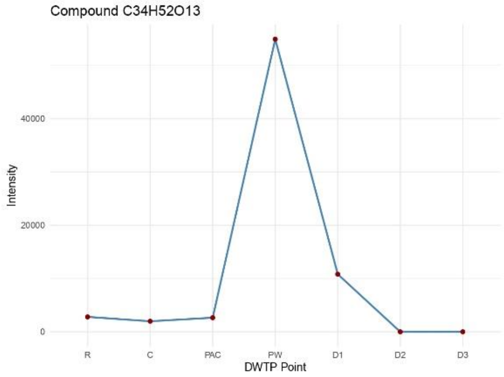
Compound C34H52O13
40000
20000
0
Intensity
R C PAC PW D1 D2 D3
DWTP Point

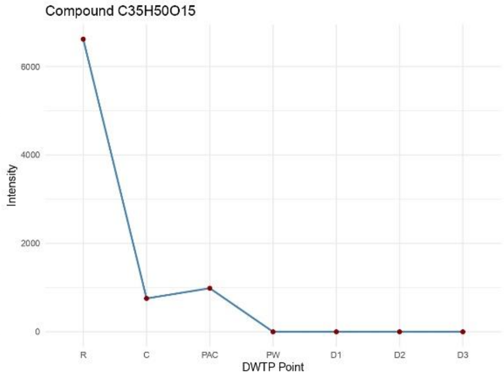
Compound C35H50O15
6000
4000
2000
0
Intensity
R C PAC PW D1 D2 D3
DWTP Point

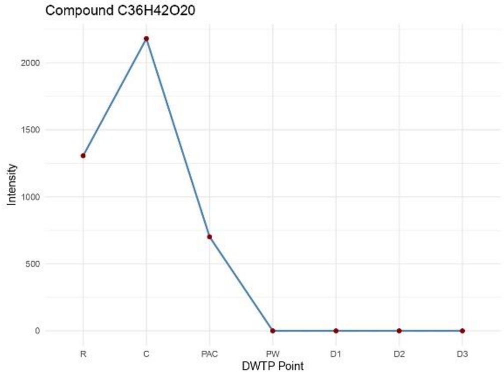
Compound C36H42O20
2000
1500
1000
500
0
Intensity
R C PAC PW D1 D2 D3
DWTP Point

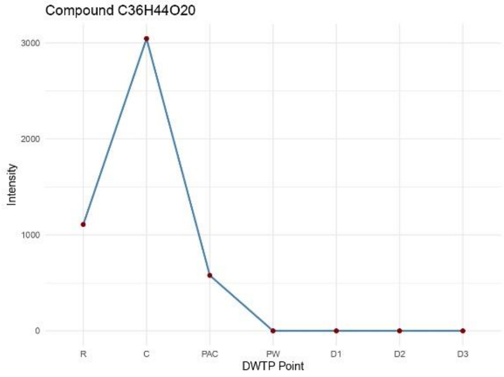
Compound C36H44O20
3000
2000
1000
0
Intensity
R C PAC PW D1 D2 D3
DWTP Point

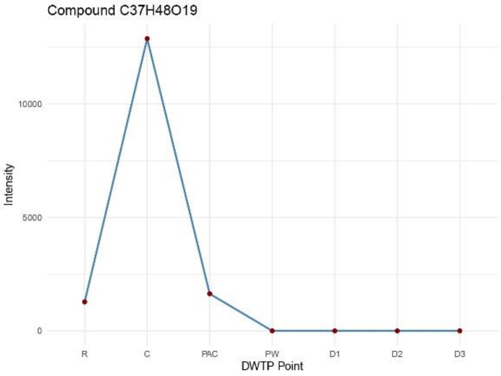
Compound C37H48O19
10000
5000
0
Intensity
R C PAC PW D1 D2 D3
DWTP Point

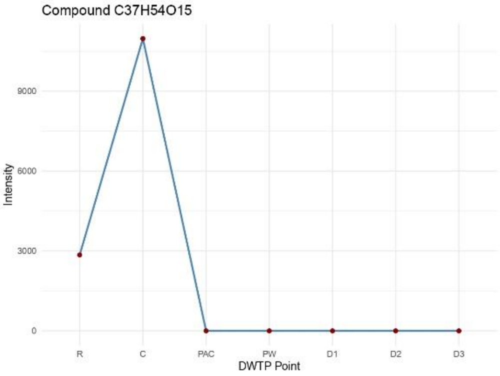
Compound C37H54O15
9000
6000
3000
0
Intensity
R C PAC PW D1 D2 D3
DWTP Point

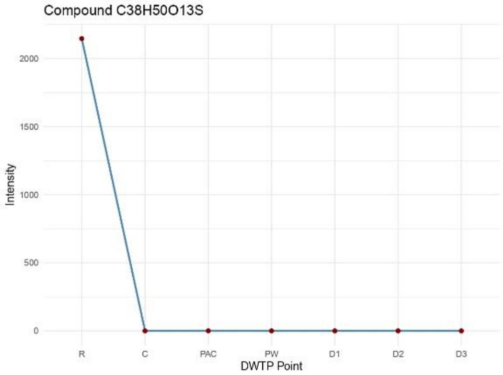
Compound C38H50O13S
2000
1500
1000
500
0
Intensity
R C PAC PW D1 D2 D3
DWTP Point

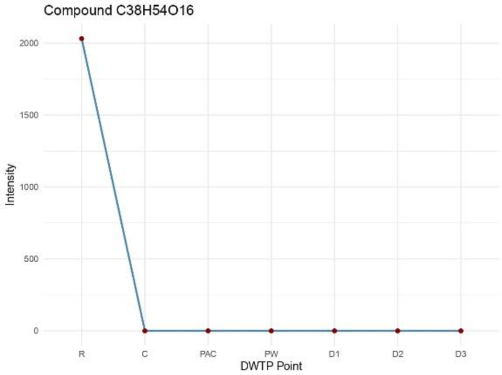
Compound C38H54O16
2000
1500
1000
500
0
Intensity
R C PAC PW D1 D2 D3
DWTP Point

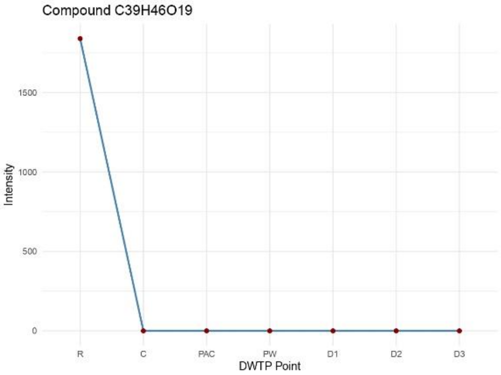
Compound C39H46O19
1500
1000
500
0
Intensity
R C PAC PW D1 D2 D3
DWTP Point

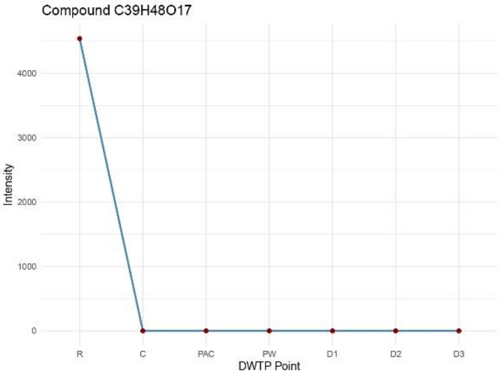
Compound C39H48O17
4000
3000
2000
1000
0
Intensity
R C PAC PW D1 D2 D3
DWTP Point

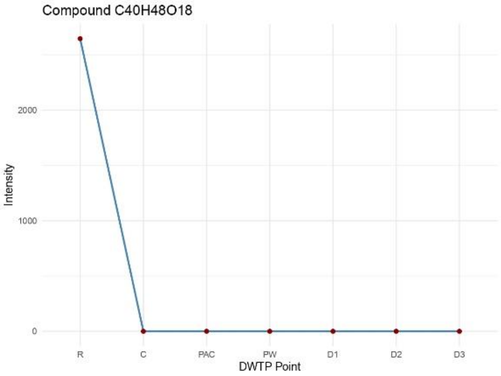
Compound C40H48O18
2000
1000
0
Intensity
R C PAC PW D1 D2 D3
DWTP Point

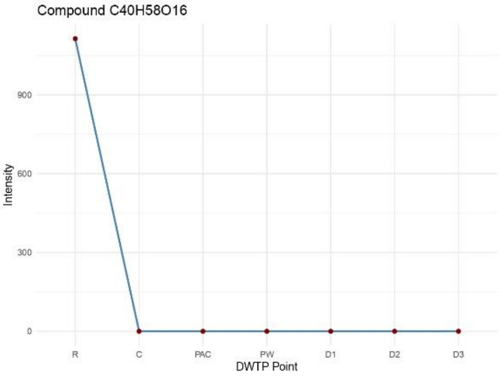
Compound C40H58O16
900
600
300
0
Intensity
R C PAC PW D1 D2 D3
DWTP Point

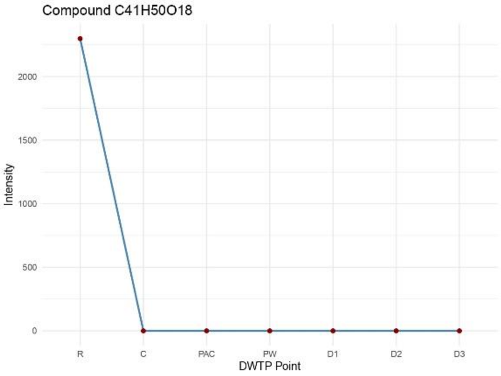
Compound C41H50O18
Intensity
0
500
1000
1500
2000
R C PAC PW D1 D2 D3
DWTP Point

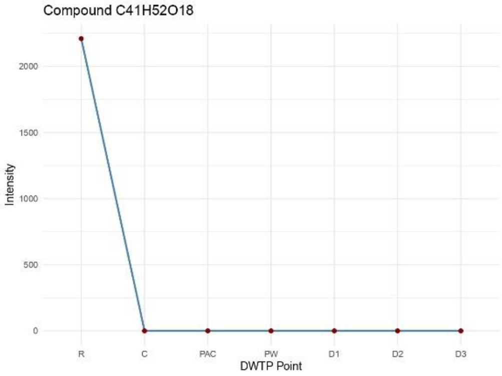
Compound C41H52O18
Intensity
0
500
1000
1500
2000
R C PAC PW D1 D2 D3
DWTP Point

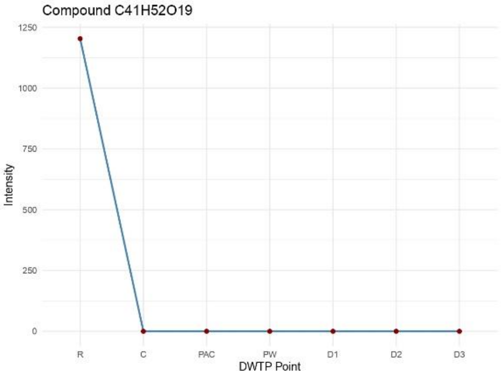
Compound C41H52O19
Intensity
0
250
500
750
1000
1250
R C PAC PW D1 D2 D3
DWTP Point

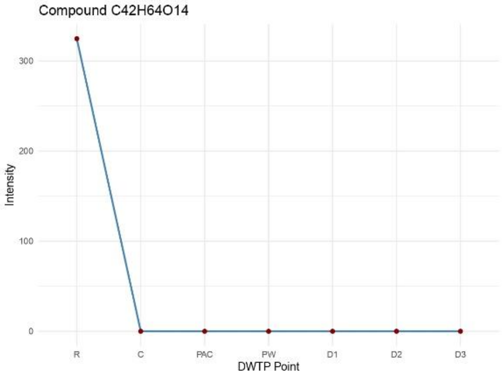
Compound C42H64O14
Intensity
0
100
200
300
R C PAC PW D1 D2 D3
DWTP Point

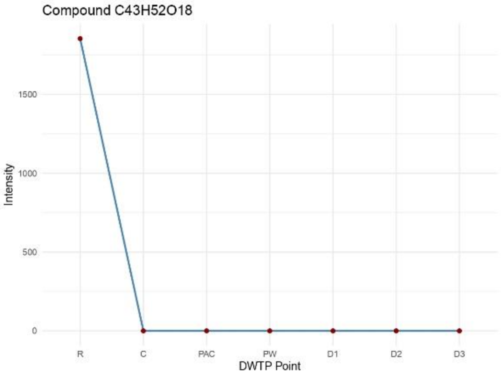
Compound C43H52O18
Intensity
0
500
1000
1500
R C PAC PW D1 D2 D3
DWTP Point

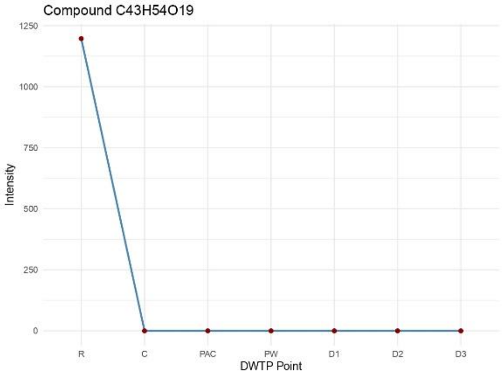
Compound C43H54O19
Intensity
0
250
500
750
1000
1250
R C PAC PW D1 D2 D3
DWTP Point

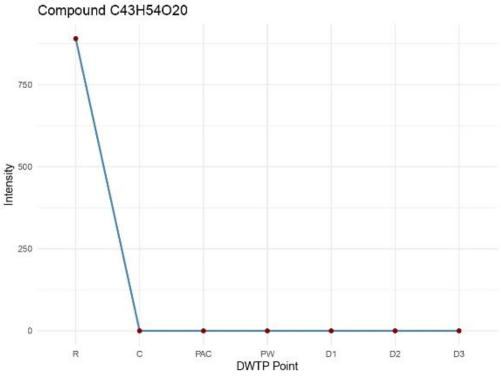
Compound C43H54O20
Intensity
0
250
500
750
R C PAC PW D1 D2 D3
DWTP Point

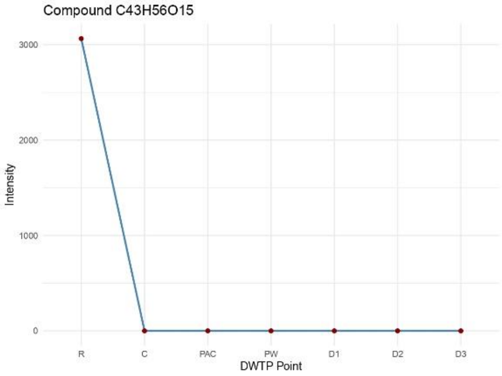
Compound C43H56O15
Intensity
0
1000
2000
3000
R C PAC PW D1 D2 D3
DWTP Point

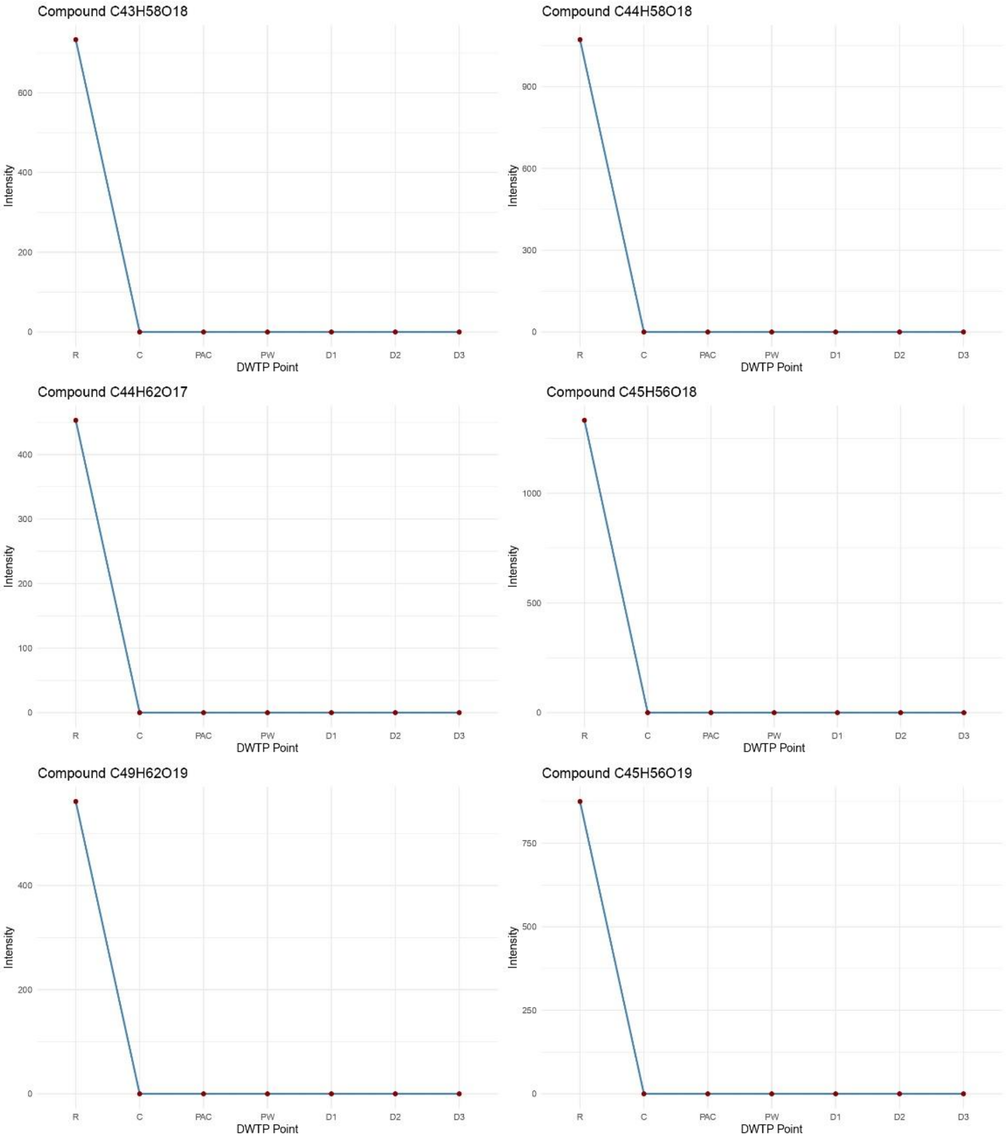
Compound C43H58O18
Intensity
600
400
200
0
R
C
PAC
PW
D1
D2
D3
DWTP Point
Compound C44H58O18
Intensity
900
600
300
0
DWTP Point
Compound C44H62O17
Intensity
400
300
200
100
0
DWTP Point
Compound C45H56O18
Intensity
1000
500
0
DWTP Point
Compound C49H62O19
Intensity
400
200
0
DWTP Point
Compound C45H56O19
Intensity
750
500
250
0
DWTP Point

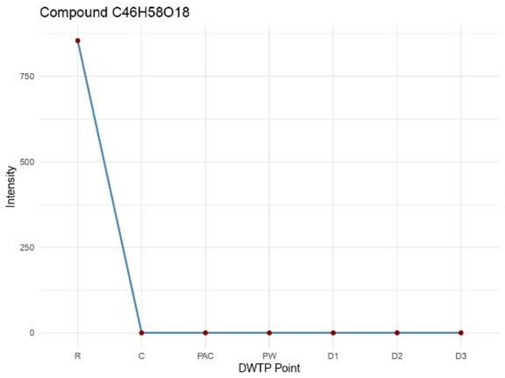
Compound C46H58O18
Intensity
750
500
250
0
R
C
PAC
PW
D1
D2
D3
DWTP Point

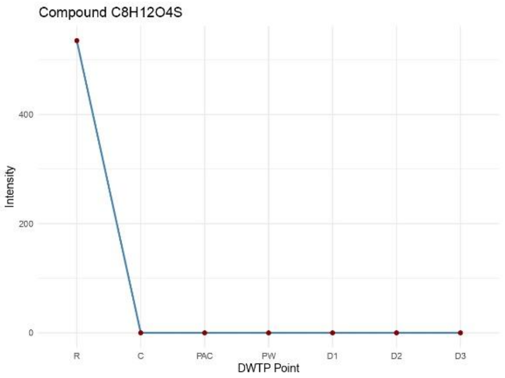
Compound C8H12O4S
Intensity
400
200
0
R
C
PAC
PW
D1
D2
D3
DWTP Point

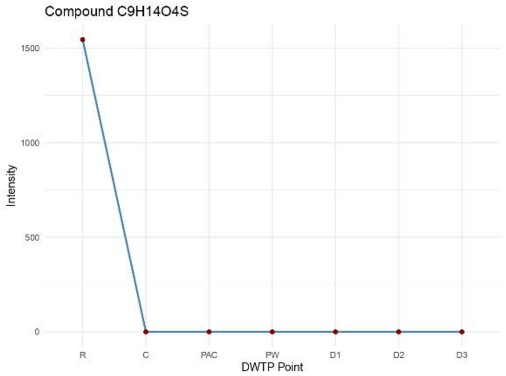
Compound C9H14O4S
Intensity
1500
1000
500
0
R
C
PAC
PW
D1
D2
D3
DWTP Point
